# Supplementary material for: Azospirillum brasilense as a Bioinoculant to Alleviate the Effects of Salinity on Quinoa Seed Germination
Source: Plants (Basel). 2025 Dec 16;14(24):3829. doi: 10.3390/plants14243829 (PMC12736936; doi:10.3390/plants14243829)
Supplement: Supplementary file 1 [file plants-14-03829-s001.zip › plants-3797206-supplementary.html]

Azospirillum brasilense as a bioinoculant to alleviate salinity effects on quinoa seed germination


## Table of contents

- 1 Project Setup
- 2 Base de datos
- 3 Import data
- 4 Statistical Analysis
  - 4.1 To determine the growth capacity of BR-11001 and BR-11002 in saline media, as a proxy for their viability and functional persistence under stress.
  - 4.2 To assess the effect of salinity gradient (0 to 450 mM NaCl) on seed germination and cotyledon emergence as indicators of bacterial efficacy
  - 4.3 To quantify seedling growth by measuring shoot and root length and dry biomass in inoculated versus non-inoculated treatments under saline and non-saline conditions
  - 4.4 To evaluate physiological responses to salinity through the activity of antioxidant enzymes (SOD, CAT, APX, GPX)
- 5 Interaction phenotipic and biochemical

# *Azospirillum brasilense* as a bioinoculant to alleviate salinity effects on quinoa seed germination

# 1 Project Setup

Code

```
library(GerminaR)
library(scales)
source('https://inkaverse.com/setup.r')
```

```
Project directory:  C:/Users/LENOVO/git/azospirillum_brasilense 
CPU cores detected:  32 
CPU cores in use:  25
```

```
─ Session info ───────────────────────────────────────────────────────────────
 setting  value
 version  R version 4.5.1 (2025-06-13 ucrt)
 os       Windows 11 x64 (build 26100)
 system   x86_64, mingw32
 ui       RTerm
 language (EN)
 collate  Spanish_Latin America.utf8
 ctype    Spanish_Latin America.utf8
 tz       America/Lima
 date     2025-08-21
 pandoc   3.4 @ C:/Program Files/RStudio/resources/app/bin/quarto/bin/tools/ (via rmarkdown)
 quarto   NA @ C:\\PROGRA~1\\Quarto\\bin\\quarto.exe

─ Packages ───────────────────────────────────────────────────────────────────
 package       * version date (UTC) lib source
 abind           1.4-8   2024-09-12 [1] CRAN (R 4.5.0)
 agricolae     * 1.3-7   2023-10-22 [1] CRAN (R 4.5.0)
 AlgDesign       1.2.1.2 2025-04-06 [1] CRAN (R 4.5.0)
 askpass         1.2.1   2024-10-04 [1] CRAN (R 4.5.0)
 boot            1.3-31  2024-08-28 [2] CRAN (R 4.5.1)
 cachem          1.1.0   2024-05-16 [1] CRAN (R 4.5.0)
 car           * 3.1-3   2024-09-27 [1] CRAN (R 4.5.0)
 carData       * 3.0-5   2022-01-06 [1] CRAN (R 4.5.0)
 cellranger      1.1.0   2016-07-27 [1] CRAN (R 4.5.0)
 cli             3.6.5   2025-04-23 [1] CRAN (R 4.5.0)
 cluster         2.1.8.1 2025-03-12 [2] CRAN (R 4.5.1)
 codetools       0.2-20  2024-03-31 [2] CRAN (R 4.5.1)
 cowplot       * 1.2.0   2025-07-07 [1] CRAN (R 4.5.1)
 curl            6.4.0   2025-06-22 [1] CRAN (R 4.5.0)
 devtools      * 2.4.5   2022-10-11 [1] CRAN (R 4.5.0)
 digest          0.6.37  2024-08-19 [1] CRAN (R 4.5.0)
 dplyr         * 1.1.4   2023-11-17 [1] CRAN (R 4.5.0)
 DT              0.33    2024-04-04 [1] CRAN (R 4.5.0)
 ellipsis        0.3.2   2021-04-29 [1] CRAN (R 4.5.0)
 emmeans       * 1.11.2  2025-07-11 [1] CRAN (R 4.5.1)
 estimability    1.5.1   2024-05-12 [1] CRAN (R 4.5.0)
 evaluate        1.0.4   2025-06-18 [1] CRAN (R 4.5.0)
 FactoMineR    * 2.12    2025-07-23 [1] CRAN (R 4.5.1)
 farver          2.1.2   2024-05-13 [1] CRAN (R 4.5.0)
 fastmap         1.2.0   2024-05-15 [1] CRAN (R 4.5.0)
 flashClust      1.01-2  2012-08-21 [1] CRAN (R 4.5.0)
 forcats       * 1.0.0   2023-01-29 [1] CRAN (R 4.5.0)
 Formula         1.2-5   2023-02-24 [1] CRAN (R 4.5.0)
 fs              1.6.6   2025-04-12 [1] CRAN (R 4.5.0)
 gargle          1.5.2   2023-07-20 [1] CRAN (R 4.5.0)
 generics        0.1.4   2025-05-09 [1] CRAN (R 4.5.0)
 GerminaR      * 2.1.5   2025-03-05 [1] CRAN (R 4.5.0)
 ggplot2       * 3.5.2   2025-04-09 [1] CRAN (R 4.5.0)
 ggrepel         0.9.6   2024-09-07 [1] CRAN (R 4.5.0)
 glue            1.8.0   2024-09-30 [1] CRAN (R 4.5.0)
 googledrive   * 2.1.1   2023-06-11 [1] CRAN (R 4.5.0)
 googlesheets4 * 1.1.1   2023-06-11 [1] CRAN (R 4.5.0)
 gtable          0.3.6   2024-10-25 [1] CRAN (R 4.5.0)
 hms             1.1.3   2023-03-21 [1] CRAN (R 4.5.0)
 htmltools       0.5.8.1 2024-04-04 [1] CRAN (R 4.5.0)
 htmlwidgets     1.6.4   2023-12-06 [1] CRAN (R 4.5.0)
 httpuv          1.6.16  2025-04-16 [1] CRAN (R 4.5.0)
 httr            1.4.7   2023-08-15 [1] CRAN (R 4.5.0)
 huito         * 0.2.5   2024-09-05 [1] CRAN (R 4.5.0)
 inti          * 0.6.7   2025-08-03 [1] local
 jsonlite        2.0.0   2025-03-27 [1] CRAN (R 4.5.0)
 knitr         * 1.50    2025-03-16 [1] CRAN (R 4.5.0)
 later           1.4.2   2025-04-08 [1] CRAN (R 4.5.0)
 lattice         0.22-7  2025-04-02 [2] CRAN (R 4.5.1)
 leaps           3.2     2024-06-10 [1] CRAN (R 4.5.0)
 lifecycle       1.0.4   2023-11-07 [1] CRAN (R 4.5.0)
 lme4          * 1.1-37  2025-03-26 [1] CRAN (R 4.5.0)
 lubridate     * 1.9.4   2024-12-08 [1] CRAN (R 4.5.0)
 magick        * 2.8.7   2025-06-06 [1] CRAN (R 4.5.0)
 magrittr        2.0.3   2022-03-30 [1] CRAN (R 4.5.0)
 MASS          * 7.3-65  2025-02-28 [2] CRAN (R 4.5.1)
 Matrix        * 1.7-3   2025-03-11 [2] CRAN (R 4.5.1)
 memoise         2.0.1   2021-11-26 [1] CRAN (R 4.5.0)
 mime            0.13    2025-03-17 [1] CRAN (R 4.5.0)
 miniUI          0.1.2   2025-04-17 [1] CRAN (R 4.5.0)
 minqa           1.2.8   2024-08-17 [1] CRAN (R 4.5.0)
 mnormt          2.1.1   2022-09-26 [1] CRAN (R 4.5.0)
 multcomp      * 1.4-28  2025-01-29 [1] CRAN (R 4.5.0)
 multcompView    0.1-10  2024-03-08 [1] CRAN (R 4.5.0)
 mvtnorm       * 1.3-3   2025-01-10 [1] CRAN (R 4.5.0)
 nlme            3.1-168 2025-03-31 [2] CRAN (R 4.5.1)
 nloptr          2.2.1   2025-03-17 [1] CRAN (R 4.5.0)
 openssl         2.3.3   2025-05-26 [1] CRAN (R 4.5.0)
 pillar          1.11.0  2025-07-04 [1] CRAN (R 4.5.1)
 pkgbuild        1.4.8   2025-05-26 [1] CRAN (R 4.5.0)
 pkgconfig       2.0.3   2019-09-22 [1] CRAN (R 4.5.0)
 pkgload         1.4.0   2024-06-28 [1] CRAN (R 4.5.0)
 profvis         0.4.0   2024-09-20 [1] CRAN (R 4.5.0)
 promises        1.3.3   2025-05-29 [1] CRAN (R 4.5.0)
 psych         * 2.5.6   2025-06-23 [1] CRAN (R 4.5.0)
 purrr         * 1.1.0   2025-07-10 [1] CRAN (R 4.5.1)
 R6              2.6.1   2025-02-15 [1] CRAN (R 4.5.0)
 rappdirs        0.3.3   2021-01-31 [1] CRAN (R 4.5.0)
 rbibutils       2.3     2024-10-04 [1] CRAN (R 4.5.0)
 RColorBrewer    1.1-3   2022-04-03 [1] CRAN (R 4.5.0)
 Rcpp            1.1.0   2025-07-02 [1] CRAN (R 4.5.1)
 Rdpack          2.6.4   2025-04-09 [1] CRAN (R 4.5.0)
 readr         * 2.1.5   2024-01-10 [1] CRAN (R 4.5.0)
 reformulas      0.4.1   2025-04-30 [1] CRAN (R 4.5.0)
 remotes         2.5.0   2024-03-17 [1] CRAN (R 4.5.0)
 RhpcBLASctl   * 0.23-42 2023-02-11 [1] CRAN (R 4.5.0)
 rlang           1.1.6   2025-04-11 [1] CRAN (R 4.5.0)
 rmarkdown       2.29    2024-11-04 [1] CRAN (R 4.5.0)
 rstudioapi      0.17.1  2024-10-22 [1] CRAN (R 4.5.0)
 sandwich        3.1-1   2024-09-15 [1] CRAN (R 4.5.0)
 scales        * 1.4.0   2025-04-24 [1] CRAN (R 4.5.0)
 scatterplot3d   0.3-44  2023-05-05 [1] CRAN (R 4.5.0)
 sessioninfo   * 1.2.3   2025-02-05 [1] CRAN (R 4.5.0)
 shiny         * 1.11.1  2025-07-03 [1] CRAN (R 4.5.1)
 showtext        0.9-7   2024-03-02 [1] CRAN (R 4.5.0)
 showtextdb      3.0     2020-06-04 [1] CRAN (R 4.5.0)
 stringi         1.8.7   2025-03-27 [1] CRAN (R 4.5.0)
 stringr       * 1.5.1   2023-11-14 [1] CRAN (R 4.5.0)
 survival      * 3.8-3   2024-12-17 [2] CRAN (R 4.5.1)
 sysfonts        0.8.9   2024-03-02 [1] CRAN (R 4.5.0)
 TH.data       * 1.1-3   2025-01-17 [1] CRAN (R 4.5.0)
 tibble        * 3.3.0   2025-06-08 [1] CRAN (R 4.5.0)
 tidyr         * 1.3.1   2024-01-24 [1] CRAN (R 4.5.0)
 tidyselect      1.2.1   2024-03-11 [1] CRAN (R 4.5.0)
 tidyverse     * 2.0.0   2023-02-22 [1] CRAN (R 4.5.1)
 timechange      0.3.0   2024-01-18 [1] CRAN (R 4.5.0)
 tzdb            0.5.0   2025-03-15 [1] CRAN (R 4.5.0)
 urlchecker      1.0.1   2021-11-30 [1] CRAN (R 4.5.0)
 usethis       * 3.1.0   2024-11-26 [1] CRAN (R 4.5.0)
 vctrs           0.6.5   2023-12-01 [1] CRAN (R 4.5.0)
 withr           3.0.2   2024-10-28 [1] CRAN (R 4.5.0)
 xfun            0.52    2025-04-02 [1] CRAN (R 4.5.0)
 xtable          1.8-4   2019-04-21 [1] CRAN (R 4.5.0)
 yaml            2.3.10  2024-07-26 [1] CRAN (R 4.5.0)
 zoo             1.8-14  2025-04-10 [1] CRAN (R 4.5.0)

 [1] C:/Users/LENOVO/AppData/Local/R/win-library/4.5
 [2] C:/Program Files/R/R-4.5.1/library
 * ── Packages attached to the search path.

──────────────────────────────────────────────────────────────────────────────
```

# 2 Base de datos

> https://docs.google.com/spreadsheets/d/1t20oZQ\_NR4zC8-gwkmV6UUMZwC24Hvh6WNJVtCXCF30/edit?gid=506947099#gid=506947099

# 3 Import data

Code

```
gs <- "https://docs.google.com/spreadsheets/d/1t20oZQ_NR4zC8-gwkmV6UUMZwC24Hvh6WNJVtCXCF30/edit?gid=506947099#gid=506947099" %>% 
  as_sheets_id()

bacterial <- gs %>%
  range_read("bacterial") %>%
  rename_with(~ iconv(.x, from = "UTF-8", to = "ASCII//TRANSLIT")) %>% 
  rename_with(~ str_replace_all(.x, "^[^a-zA-Z0-9]+|[^a-zA-Z0-9]+$", "")) %>% 
  rename_with(~ tolower(gsub("[^[:alnum:]_]", "_", .x))) %>% 
  mutate(across(1:repetition, ~ as.factor(.))) %>% 
  dplyr::select(where(~ mean(!is.na(.)) >= 0.1))
  
bacterial %>% str()
## tibble [168 × 5] (S3: tbl_df/tbl/data.frame)
##  $ strains         : Factor w/ 6 levels "BR-11001","BR-11002",..: 1 1 1 1 1 1 1 1 1 1 ...
##  $ nacl            : Factor w/ 7 levels "0","150","300",..: 1 1 1 1 2 2 2 2 3 3 ...
##  $ repetition      : Factor w/ 4 levels "R1","R2","R3",..: 1 2 3 4 1 2 3 4 1 2 ...
##  $ od_600_nm       : num [1:168] 1.29 1.3 1.29 1.29 1.3 1.27 1.31 1.27 1.15 1.12 ...
##  $ bacterial_growth: num [1:168] 1558000000 1570000000 1558000000 1558000000 1570000000 ...

bacterial %>% web_table()
```

Code

```
germ <- gs %>%
  range_read("radicula") %>%
  rename_with(~ iconv(.x, from = "UTF-8", to = "ASCII//TRANSLIT")) %>% 
  rename_with(~ str_replace_all(.x, "^[^a-zA-Z0-9]+|[^a-zA-Z0-9]+$", "")) %>% 
  rename_with(~ tolower(gsub("[^[:alnum:]_]", "_", .x))) %>% 
  mutate(across(1:nacl, ~ as.factor(.))) %>% 
  dplyr::select(where(~ mean(!is.na(.)) >= 0.1))
  
germ %>% str()
## tibble [48 × 15] (S3: tbl_df/tbl/data.frame)
##  $ repetition: Factor w/ 4 levels "R1","R2","R3",..: 1 2 3 4 1 2 3 4 1 2 ...
##  $ strains   : Factor w/ 3 levels "BR-11001","BR-11002",..: 3 3 3 3 3 3 3 3 3 3 ...
##  $ nacl      : Factor w/ 4 levels "0","150","300",..: 1 1 1 1 2 2 2 2 3 3 ...
##  $ seeds     : num [1:48] 50 50 50 50 50 50 50 50 50 50 ...
##  $ d0        : num [1:48] 0 0 0 0 0 0 0 0 0 0 ...
##  $ d1        : num [1:48] 44 43 42 44 37 39 38 36 6 6 ...
##  $ d2        : num [1:48] 3 0 3 3 2 1 4 3 21 24 ...
##  $ d3        : num [1:48] 0 2 0 0 2 2 2 3 17 16 ...
##  $ d4        : num [1:48] 2 3 3 1 4 3 2 4 2 0 ...
##  $ d5        : num [1:48] 0 2 2 2 5 4 2 2 1 1 ...
##  $ d6        : num [1:48] 0 0 0 0 0 0 0 0 0 0 ...
##  $ d7        : num [1:48] 0 0 0 0 0 1 1 2 0 0 ...
##  $ d8        : num [1:48] 1 0 0 0 0 0 1 0 0 0 ...
##  $ d9        : num [1:48] 0 0 0 0 0 0 0 0 0 0 ...
##  $ d10       : num [1:48] 0 0 0 0 0 0 0 0 1 0 ...

germ %>% web_table()
```

Code

```
cot <- gs %>%
  range_read("germination") %>%
  rename_with(~ iconv(.x, from = "UTF-8", to = "ASCII//TRANSLIT")) %>% 
  rename_with(~ str_replace_all(.x, "^[^a-zA-Z0-9]+|[^a-zA-Z0-9]+$", "")) %>% 
  rename_with(~ tolower(gsub("[^[:alnum:]_]", "_", .x))) %>% 
  mutate(across(1:nacl, ~ as.factor(.))) %>% 
  dplyr::select(where(~ mean(!is.na(.)) >= 0.1))
  
cot %>% str()
## tibble [48 × 15] (S3: tbl_df/tbl/data.frame)
##  $ repetition: Factor w/ 4 levels "R1","R2","R3",..: 1 2 3 4 1 2 3 4 1 2 ...
##  $ strains   : Factor w/ 3 levels "BR-11001","BR-11002",..: 3 3 3 3 3 3 3 3 3 3 ...
##  $ nacl      : Factor w/ 4 levels "0","150","300",..: 1 1 1 1 2 2 2 2 3 3 ...
##  $ seeds     : num [1:48] 50 50 50 50 50 50 50 50 50 50 ...
##  $ d0        : num [1:48] 0 0 0 0 0 0 0 0 0 0 ...
##  $ d1        : num [1:48] 0 0 0 0 0 0 0 0 0 0 ...
##  $ d2        : num [1:48] 0 0 0 0 0 0 0 0 0 0 ...
##  $ d3        : num [1:48] 11 11 11 9 0 2 5 5 0 0 ...
##  $ d4        : num [1:48] 21 17 16 19 14 13 11 9 0 0 ...
##  $ d5        : num [1:48] 1 3 4 4 7 5 7 8 9 7 ...
##  $ d6        : num [1:48] 4 8 9 5 10 6 4 5 9 6 ...
##  $ d7        : num [1:48] 7 8 8 8 5 3 3 7 9 15 ...
##  $ d8        : num [1:48] 6 2 1 5 6 7 7 4 2 2 ...
##  $ d9        : num [1:48] 0 0 0 0 5 9 7 8 1 2 ...
##  $ d10       : num [1:48] 0 0 0 0 2 4 6 3 3 3 ...

cot %>% web_table()
```

Code

```
growth <- gs %>%
  range_read("growth") %>%
  rename_with(~ iconv(.x, from = "UTF-8", to = "ASCII//TRANSLIT")) %>% 
  rename_with(~ str_replace_all(.x, "^[^a-zA-Z0-9]+|[^a-zA-Z0-9]+$", "")) %>% 
  rename_with(~ tolower(gsub("[^[:alnum:]_]", "_", .x))) %>% 
  mutate(across(1:repetition, ~ as.factor(.))) %>% 
  dplyr::select(where(~ mean(!is.na(.)) >= 0.1))
  
growth %>% str()
## tibble [48 × 14] (S3: tbl_df/tbl/data.frame)
##  $ strains      : Factor w/ 3 levels "BR-11001","BR-11002",..: 3 3 3 3 3 3 3 3 3 3 ...
##  $ nacl         : Factor w/ 4 levels "0","150","300",..: 1 1 1 1 2 2 2 2 3 3 ...
##  $ repetition   : Factor w/ 4 levels "R1","R2","R3",..: 1 2 3 4 1 2 3 4 1 2 ...
##  $ shoot_length : num [1:48] 4.74 4.68 4.82 4.84 4.75 ...
##  $ root_length  : num [1:48] 3.12 2.97 3.07 3.05 3.04 ...
##  $ fresh_weight : num [1:48] 0.343 0.343 0.343 0.343 0.343 ...
##  $ dry_weight   : num [1:48] 0.0268 0.0264 0.0266 0.0257 0.0271 ...
##  $ ms_porc      : num [1:48] 7.81 7.7 7.75 7.5 7.89 ...
##  $ humidity_cont: num [1:48] 0.316 0.316 0.317 0.317 0.316 ...
##  $ humidity_porc: num [1:48] 92.2 92.3 92.3 92.5 92.1 ...
##  $ sod          : num [1:48] 22.9 24 24.1 24.9 34.9 ...
##  $ cat          : num [1:48] 13.1 13.8 14.4 15.3 20.8 ...
##  $ apx          : num [1:48] 8.5 8.99 9.61 10.34 13.7 ...
##  $ gpx          : num [1:48] 5.9 6.54 7.67 8.48 9.3 ...

growth %>% web_table()
```

Code

```
# https://colorbrewer2.org/
```

# 4 Statistical Analysis

## 4.1 To determine the growth capacity of BR-11001 and BR-11002 in saline media, as a proxy for their viability and functional persistence under stress.

Code

```
dtx <- bacterial 
# trait <- "bacterial_growth"

dtx %>% str()
## tibble [168 × 5] (S3: tbl_df/tbl/data.frame)
##  $ strains         : Factor w/ 6 levels "BR-11001","BR-11002",..: 1 1 1 1 1 1 1 1 1 1 ...
##  $ nacl            : Factor w/ 7 levels "0","150","300",..: 1 1 1 1 2 2 2 2 3 3 ...
##  $ repetition      : Factor w/ 4 levels "R1","R2","R3",..: 1 2 3 4 1 2 3 4 1 2 ...
##  $ od_600_nm       : num [1:168] 1.29 1.3 1.29 1.29 1.3 1.27 1.31 1.27 1.15 1.12 ...
##  $ bacterial_growth: num [1:168] 1558000000 1570000000 1558000000 1558000000 1570000000 ...

rs <- xfun::cache_rds({
  
  traits <- names(dtx)[4:length(dtx)]
  
  results <- traits %>% set_names() %>% map( \(trait) {
    
    # Liberar memoria
    gc()
    
    # Definir modelo
    mdf <- paste(trait, "0 + strains*nacl + (1|repetition)", sep = " ~ ") 
    
    # Mostrar qué rasgo se analiza
    cat("\n\n## Análisis para: ", trait, "\n\n")
    
    
    cat("\n\n### Diagnostico de modelo\n\n")
    
    # Remover outliers (asumo que tienes esta función personalizada)
    md <- dtx %>% 
      remove_outliers(as.formula(mdf),
                      plot_diag = TRUE,
                      drop_na = TRUE)
    
    md$diagplot %>% print()
    
    if (nrow(md$outliers) > 0) {
    cat("> Outliers detectados\n")
    md$outliers %>% kable() %>% print()
    } else {
    cat("> No se detectaron outliers\n")
    }
    
    mdc <- md$model$clean
    
    cat("\n\n### ANOVA tipo III\n\n")
    
    Anova(mdc, type = 3, test.statistic = "F") %>%  print()
    
    cat("\n\n### Comparaciones múltiples (Emmeans)\n\n")
    
    # Comparaciones múltiples into the groups
    mc_in <- emmeans(mdc, ~ strains|nacl, type = "response") %>% 
      cld(Letters = letters, reversed = TRUE) %>% 
      mutate(across(".group", trimws)) %>% 
      mutate(across(".group", tolower)) %>% 
      rename(sig_in = ".group")
    
    mc_ou <- emmeans(mdc, ~ nacl|strains, type = "response") %>% 
      cld(Letters = letters, reversed = TRUE) %>% 
      mutate(across(".group", trimws)) %>% 
      mutate(across(".group", toupper)) %>% 
      rename(sig_ou = ".group")
    
    mc <- merge(mc_in, mc_ou) %>% 
      unite(col = "sig", c("sig_in", "sig_ou"), sep = "")
    
    mc %>% kable() %>% print()
    
    cat("\n\n### Gráfico resumen\n\n")
    
    plot <- mc %>% 
      plot_smr(type = "line",
               x = "nacl",
               y = "emmean"
               , group = "strains"
               , ylab = trait
               , sig = "sig"
               , error = "SE") +
      scale_color_manual(values = c("#fbb4ae", "#b3cde3", "#ccebc5"
                                    , "#decbe4", "#fed9a6", "#ffffcc"))
    
    plot %>% print()
    
    list(mc = mc,
         plot = plot
         )
  })
  
  results
  
}, file = "result.rds", rerun = T)
## 
## 
## ## Análisis para:  od_600_nm 
## 
## 
## 
## ### Diagnostico de modelo
```

```
## > No se detectaron outliers
## 
## 
## ### ANOVA tipo III
## 
## Analysis of Deviance Table (Type III Wald F tests with Kenward-Roger df)
## 
## Response: od_600_nm
##                      F Df Df.res                Pr(>F)    
## strains      21795.114  6 121.64 < 0.00000000000000022 ***
## nacl          1645.651  6 123.00 < 0.00000000000000022 ***
## strains:nacl    18.121 30 123.00 < 0.00000000000000022 ***
## ---
## Signif. codes:  0 '***' 0.001 '**' 0.01 '*' 0.05 '.' 0.1 ' ' 1
## 
## 
## ### Comparaciones múltiples (Emmeans)
## 
## 
## 
## |strains  |nacl | emmean|       SE|  df|  lower.CL|  upper.CL|sig |
## |:--------|:----|------:|--------:|---:|---------:|---------:|:---|
## |BR-11001 |0    | 1.2925| 0.008168| 126| 1.2763358| 1.3086642|bA  |
## |BR-11001 |150  | 1.2875| 0.008168| 126| 1.2713358| 1.3036642|bA  |
## |BR-11001 |300  | 1.1250| 0.008168| 126| 1.1088358| 1.1411642|bB  |
## |BR-11001 |450  | 0.8825| 0.008168| 126| 0.8663358| 0.8986642|bC  |
## |BR-11001 |600  | 0.7000| 0.008168| 126| 0.6838358| 0.7161642|bD  |
## |BR-11001 |750  | 0.6000| 0.008168| 126| 0.5838358| 0.6161642|bE  |
## |BR-11001 |900  | 0.4825| 0.008168| 126| 0.4663358| 0.4986642|bF  |
## |BR-11002 |0    | 1.4700| 0.008168| 126| 1.4538358| 1.4861642|aA  |
## |BR-11002 |150  | 1.4850| 0.008168| 126| 1.4688358| 1.5011642|aA  |
## |BR-11002 |300  | 1.2050| 0.008168| 126| 1.1888358| 1.2211642|aB  |
## |BR-11002 |450  | 1.0025| 0.008168| 126| 0.9863358| 1.0186642|aC  |
## |BR-11002 |600  | 0.7875| 0.008168| 126| 0.7713358| 0.8036642|aD  |
## |BR-11002 |750  | 0.7025| 0.008168| 126| 0.6863358| 0.7186642|aE  |
## |BR-11002 |900  | 0.5625| 0.008168| 126| 0.5463358| 0.5786642|aF  |
## |BR-11003 |0    | 0.9525| 0.008168| 126| 0.9363358| 0.9686642|eA  |
## |BR-11003 |150  | 0.8400| 0.008168| 126| 0.8238358| 0.8561642|eB  |
## |BR-11003 |300  | 0.7725| 0.008168| 126| 0.7563358| 0.7886642|fC  |
## |BR-11003 |450  | 0.6125| 0.008168| 126| 0.5963358| 0.6286642|eD  |
## |BR-11003 |600  | 0.4200| 0.008168| 126| 0.4038358| 0.4361642|eE  |
## |BR-11003 |750  | 0.2650| 0.008168| 126| 0.2488358| 0.2811642|eF  |
## |BR-11003 |900  | 0.1750| 0.008168| 126| 0.1588358| 0.1911642|eG  |
## |BR-11004 |0    | 1.1825| 0.008168| 126| 1.1663358| 1.1986642|cA  |
## |BR-11004 |150  | 1.1775| 0.008168| 126| 1.1613358| 1.1936642|cA  |
## |BR-11004 |300  | 1.0125| 0.008168| 126| 0.9963358| 1.0286642|cB  |
## |BR-11004 |450  | 0.7925| 0.008168| 126| 0.7763358| 0.8086642|cC  |
## |BR-11004 |600  | 0.5900| 0.008168| 126| 0.5738358| 0.6061642|cD  |
## |BR-11004 |750  | 0.4875| 0.008168| 126| 0.4713358| 0.5036642|cE  |
## |BR-11004 |900  | 0.3675| 0.008168| 126| 0.3513358| 0.3836642|cF  |
## |BR-11005 |0    | 1.1550| 0.008168| 126| 1.1388358| 1.1711642|cdA |
## |BR-11005 |150  | 1.1575| 0.008168| 126| 1.1413358| 1.1736642|cA  |
## |BR-11005 |300  | 0.9775| 0.008168| 126| 0.9613358| 0.9936642|dB  |
## |BR-11005 |450  | 0.8000| 0.008168| 126| 0.7838358| 0.8161642|cC  |
## |BR-11005 |600  | 0.5850| 0.008168| 126| 0.5688358| 0.6011642|cdD |
## |BR-11005 |750  | 0.4475| 0.008168| 126| 0.4313358| 0.4636642|dE  |
## |BR-11005 |900  | 0.2950| 0.008168| 126| 0.2788358| 0.3111642|dF  |
## |BR-11006 |0    | 1.1250| 0.008168| 126| 1.1088358| 1.1411642|dA  |
## |BR-11006 |150  | 1.1200| 0.008168| 126| 1.1038358| 1.1361642|dA  |
## |BR-11006 |300  | 0.9275| 0.008168| 126| 0.9113358| 0.9436642|eB  |
## |BR-11006 |450  | 0.7525| 0.008168| 126| 0.7363358| 0.7686642|dC  |
## |BR-11006 |600  | 0.5550| 0.008168| 126| 0.5388358| 0.5711642|dD  |
## |BR-11006 |750  | 0.4375| 0.008168| 126| 0.4213358| 0.4536642|dE  |
## |BR-11006 |900  | 0.2650| 0.008168| 126| 0.2488358| 0.2811642|dF  |
## 
## 
## ### Gráfico resumen
```

```
## 
## 
## ## Análisis para:  bacterial_growth 
## 
## 
## 
## ### Diagnostico de modelo
```

```
## > No se detectaron outliers
## 
## 
## ### ANOVA tipo III
## 
## Analysis of Deviance Table (Type III Wald F tests with Kenward-Roger df)
## 
## Response: bacterial_growth
##                      F Df Df.res                Pr(>F)    
## strains      22094.579  6 121.64 < 0.00000000000000022 ***
## nacl          1645.651  6 123.00 < 0.00000000000000022 ***
## strains:nacl    18.121 30 123.00 < 0.00000000000000022 ***
## ---
## Signif. codes:  0 '***' 0.001 '**' 0.01 '*' 0.05 '.' 0.1 ' ' 1
## 
## 
## ### Comparaciones múltiples (Emmeans)
## 
## 
## 
## |strains  |nacl |     emmean|      SE|  df|   lower.CL|   upper.CL|sig |
## |:--------|:----|----------:|-------:|---:|----------:|----------:|:---|
## |BR-11001 |0    | 1561000000| 9801603| 126| 1541602915| 1580397085|bA  |
## |BR-11001 |150  | 1555000000| 9801603| 126| 1535602915| 1574397085|bA  |
## |BR-11001 |300  | 1360000000| 9801603| 126| 1340602915| 1379397085|bB  |
## |BR-11001 |450  | 1069000000| 9801603| 126| 1049602915| 1088397085|bC  |
## |BR-11001 |600  |  850000000| 9801603| 126|  830602915|  869397085|bD  |
## |BR-11001 |750  |  730000000| 9801603| 126|  710602915|  749397085|bE  |
## |BR-11001 |900  |  589000000| 9801603| 126|  569602915|  608397085|bF  |
## |BR-11002 |0    | 1774000000| 9801603| 126| 1754602915| 1793397085|aA  |
## |BR-11002 |150  | 1792000000| 9801603| 126| 1772602915| 1811397085|aA  |
## |BR-11002 |300  | 1456000000| 9801603| 126| 1436602915| 1475397085|aB  |
## |BR-11002 |450  | 1213000000| 9801603| 126| 1193602915| 1232397085|aC  |
## |BR-11002 |600  |  955000000| 9801603| 126|  935602915|  974397085|aD  |
## |BR-11002 |750  |  853000000| 9801603| 126|  833602915|  872397085|aE  |
## |BR-11002 |900  |  685000000| 9801603| 126|  665602915|  704397085|aF  |
## |BR-11003 |0    | 1153000000| 9801603| 126| 1133602915| 1172397085|eA  |
## |BR-11003 |150  | 1018000000| 9801603| 126|  998602915| 1037397085|eB  |
## |BR-11003 |300  |  937000000| 9801603| 126|  917602915|  956397085|fC  |
## |BR-11003 |450  |  745000000| 9801603| 126|  725602915|  764397085|eD  |
## |BR-11003 |600  |  514000000| 9801603| 126|  494602915|  533397085|eE  |
## |BR-11003 |750  |  328000000| 9801603| 126|  308602915|  347397085|eF  |
## |BR-11003 |900  |  220000000| 9801603| 126|  200602915|  239397085|eG  |
## |BR-11004 |0    | 1429000000| 9801603| 126| 1409602915| 1448397085|cA  |
## |BR-11004 |150  | 1423000000| 9801603| 126| 1403602915| 1442397085|cA  |
## |BR-11004 |300  | 1225000000| 9801603| 126| 1205602915| 1244397085|cB  |
## |BR-11004 |450  |  961000000| 9801603| 126|  941602915|  980397085|cC  |
## |BR-11004 |600  |  718000000| 9801603| 126|  698602915|  737397085|cD  |
## |BR-11004 |750  |  595000000| 9801603| 126|  575602915|  614397085|cE  |
## |BR-11004 |900  |  451000000| 9801603| 126|  431602915|  470397085|cF  |
## |BR-11005 |0    | 1396000000| 9801603| 126| 1376602915| 1415397085|cdA |
## |BR-11005 |150  | 1399000000| 9801603| 126| 1379602915| 1418397085|cA  |
## |BR-11005 |300  | 1183000000| 9801603| 126| 1163602915| 1202397085|dB  |
## |BR-11005 |450  |  970000000| 9801603| 126|  950602915|  989397085|cC  |
## |BR-11005 |600  |  712000000| 9801603| 126|  692602915|  731397085|cdD |
## |BR-11005 |750  |  547000000| 9801603| 126|  527602915|  566397085|dE  |
## |BR-11005 |900  |  364000000| 9801603| 126|  344602915|  383397085|dF  |
## |BR-11006 |0    | 1360000000| 9801603| 126| 1340602915| 1379397085|dA  |
## |BR-11006 |150  | 1354000000| 9801603| 126| 1334602915| 1373397085|dA  |
## |BR-11006 |300  | 1123000000| 9801603| 126| 1103602915| 1142397085|eB  |
## |BR-11006 |450  |  913000000| 9801603| 126|  893602915|  932397085|dC  |
## |BR-11006 |600  |  676000000| 9801603| 126|  656602915|  695397085|dD  |
## |BR-11006 |750  |  535000000| 9801603| 126|  515602915|  554397085|dE  |
## |BR-11006 |900  |  328000000| 9801603| 126|  308602915|  347397085|dF  |
## 
## 
## ### Gráfico resumen
```

Code

```
plots <- list(rs$od_600_nm$plot +
                scale_y_continuous(limits = c(0, 1.75)
                                  , breaks = seq(0, 200, by = 0.25)) +
                labs(x = ""
                     , y = expression(OD[600]~"(nm)")
                     ) #+
                # theme(legend.position = "none"
                #       , axis.text.x = element_blank()
                #       , strip.text = element_blank()
                #       )
              , rs$bacterial_growth$plot +
                scale_y_log10(
                  limits = c(2e8, 20e8),
                  breaks = 10^seq(8, 20, by = 0.1),
                  labels = trans_format("log10", math_format(10^.x))
                  ) +
                labs(x = ""
                     , y = "Bacterial growth"
                     ) +
                theme(legend.position = "none"
                      # , axis.text.x = element_blank()
                      # , strip.text = element_blank()
                      )
              )

legend <- get_plot_component(
  rs$bacterial_growth$plot +
    guides(color = guide_legend(nrow = 1)) +  # Esto fuerza una sola línea
    theme(
      legend.position = "bottom",
      legend.direction = "horizontal",
      legend.box = "horizontal"
    ),
  "guide-box",
  return_all = TRUE
)

fgrids <- plots %>% 
  cowplot::plot_grid(plotlist = ., ncol = 1, labels = "auto") 

export <-  list(legend[[3]], fgrids) %>% 
  cowplot::plot_grid(plotlist = .
                     , ncol = 1
                     , rel_heights = c(0.1, 1))
  
plot <- plots[[1]] %>% 
  ggsave2(plot = ., "manuscript/Figure-01.jpg", width = 16, height = 15
         , units = "cm")

plot %>% include_graphics()
```

Code

```
plots[[1]] %>% 
  ggsave2(plot = ., "manuscript/Figure-01.eps", width = 16, height = 15
         , units = "cm")
```

## 4.2 To assess the effect of salinity gradient (0 to 450 mM NaCl) on seed germination and cotyledon emergence as indicators of bacterial efficacy

Code

```
circular_crop <- function(img) {
  # Obtener dimensiones de la imagen
  info <- image_info(img)
  size <- min(info$width, info$height) # Usar la menor dimensión para hacer la imagen cuadrada
  
  # Recortar la imagen a un tamaño cuadrado centrado
  img_square <- image_crop(img, paste0(size, "x", size, "+0+0"))

  # Crear una máscara circular
  mask <- image_blank(size, size, color = "none") %>% # Fondo transparente
    image_draw()
  grid::grid.circle(r = 0.4, gp = grid::gpar(fill = "black", col = NA)) # Círculo negro sobre fondo transparente
  dev.off()

  # Aplicar la máscara (recortar la imagen en un círculo)
  img_circular <- image_composite(img_square, mask, operator = "CopyOpacity")

  # Crear un fondo negro del mismo tamaño
  background <- image_blank(size, size, color = "black")

  # Poner la imagen circular sobre el fondo negro
  img_final <- image_composite(background, img_circular, operator = "Over")

  return(img_final)
}

plates <- list.files(path = "photos/"
                  , full.names = T
                  , recursive = T
                  ) 

lbls <- plates %>% 
  gsub(".*_(.*)", "\\1", .) %>% 
  gsub("^(\\d+)-days", "\\1 days", .) %>% 
  gsub(".png", "", .) 

lbls %>% dput()
## c("BR-11001", "5 days", "8 days", "10 days", "BR-11002", "5 days", 
## "8 days", "10 days", "Control", "5 days", "8 days", "10 days"
## )

grids <- plates %>% 
  map( ~ image_read(.)) %>% 
  # map( ~ circular_crop(.)) %>%
  map( ~ image_transparent(., "black")) %>%
  map( ~ image_ggplot(.))

rw1 <- grids[9:12] %>% 
  plot_grid(plotlist = ., ncol = 4
            , labels = c("", "5 days", "8 days", "10 days")
            , label_x = 0.15
            )

rw2 <- grids[1:4] %>% 
  plot_grid(plotlist = ., ncol = 4)

rw3 <- grids[5:8] %>% 
  plot_grid(plotlist = ., ncol = 4)


plots <- list(rw1, rw2, rw3) %>% 
  plot_grid(plotlist = ., nrow = 3
            , labels = c("Control", "BR-11001", "BR-11002")
            , hjust = 0
            ) %>% 
  ggsave2(plot = ., "manuscript/Figure-02.jpg"
          , width = 25, height = 22, units = "cm")
  
plot %>% include_graphics()
```

Code

```
list(rw1, rw2, rw3) %>% 
  plot_grid(plotlist = ., nrow = 3
            , labels = c("Control", "BR-11001", "BR-11002")
            , hjust = 0
            ) %>% 
  ggsave2(plot = ., "manuscript/Figure-02.eps"
          , width = 25, height = 22, units = "cm")
```

Code

```
# germination analysis (ten variables)

gsm <- germ %>% 
  as.data.frame() %>% 
  ger_summary(SeedN = "seeds"
              , evalName = "d"
              , data = .
              ) %>% 
  dplyr::select(1:nacl, grp, mgt, unc, syn)

gsm %>% kable()
```

| repetition | strains | nacl | grp | mgt | unc | syn |
| --- | --- | --- | --- | --- | --- | --- |
| R1 | Control | 0 | 100 | 1.320000 | 0.7044586 | 0.7755102 |
| R2 | Control | 0 | 100 | 1.420000 | 0.8021708 | 0.7412245 |
| R3 | Control | 0 | 100 | 1.400000 | 0.8841141 | 0.7085714 |
| R4 | Control | 0 | 100 | 1.280000 | 0.7044586 | 0.7755102 |
| R1 | Control | 150 | 100 | 1.760000 | 1.3166679 | 0.5583673 |
| R2 | Control | 150 | 100 | 1.720000 | 1.2261447 | 0.6130612 |
| R3 | Control | 150 | 100 | 1.700000 | 1.3754313 | 0.5812245 |
| R4 | Control | 150 | 100 | 1.820000 | 1.4913147 | 0.5257143 |
| R1 | Control | 300 | 96 | 2.541667 | 1.8508936 | 0.3209220 |
| R2 | Control | 300 | 94 | 2.276596 | 1.5216341 | 0.3802035 |
| R3 | Control | 300 | 96 | 2.562500 | 1.5333514 | 0.3785461 |
| R4 | Control | 300 | 96 | 2.458333 | 1.8861784 | 0.2969858 |
| R1 | Control | 450 | 56 | 5.714286 | 2.9686427 | 0.1111111 |
| R2 | Control | 450 | 52 | 5.538462 | 2.8552794 | 0.1138462 |
| R3 | Control | 450 | 52 | 6.115385 | 2.8073905 | 0.1200000 |
| R4 | Control | 450 | 50 | 5.600000 | 2.7932697 | 0.1233333 |
| R1 | BR-11001 | 0 | 100 | 1.140000 | 0.5411884 | 0.8130612 |
| R2 | BR-11001 | 0 | 100 | 1.180000 | 0.5221792 | 0.8457143 |
| R3 | BR-11001 | 0 | 100 | 1.140000 | 0.5411884 | 0.8130612 |
| R4 | BR-11001 | 0 | 100 | 1.140000 | 0.5221792 | 0.8457143 |
| R1 | BR-11001 | 150 | 100 | 1.720000 | 1.2510470 | 0.6114286 |
| R2 | BR-11001 | 150 | 100 | 1.800000 | 1.2112160 | 0.6408163 |
| R3 | BR-11001 | 150 | 100 | 1.700000 | 1.3212425 | 0.6106122 |
| R4 | BR-11001 | 150 | 100 | 1.580000 | 1.1312160 | 0.6440816 |
| R1 | BR-11001 | 300 | 98 | 2.020408 | 1.8611296 | 0.3027211 |
| R2 | BR-11001 | 300 | 100 | 2.340000 | 1.6586919 | 0.3232653 |
| R3 | BR-11001 | 300 | 98 | 2.081633 | 1.7188777 | 0.3154762 |
| R4 | BR-11001 | 300 | 98 | 2.061224 | 1.5385791 | 0.3418367 |
| R1 | BR-11001 | 450 | 76 | 5.236842 | 2.8813999 | 0.1379801 |
| R2 | BR-11001 | 450 | 78 | 5.128205 | 2.7550705 | 0.1457490 |
| R3 | BR-11001 | 450 | 80 | 5.425000 | 2.9402410 | 0.1243590 |
| R4 | BR-11001 | 450 | 80 | 5.075000 | 3.0495818 | 0.1179487 |
| R1 | BR-11002 | 0 | 100 | 1.100000 | 0.4689956 | 0.8163265 |
| R2 | BR-11002 | 0 | 100 | 1.080000 | 0.4021792 | 0.8497959 |
| R3 | BR-11002 | 0 | 100 | 1.120000 | 0.5293609 | 0.7844898 |
| R4 | BR-11002 | 0 | 100 | 1.140000 | 0.5842388 | 0.7542857 |
| R1 | BR-11002 | 150 | 100 | 1.440000 | 0.8421708 | 0.7404082 |
| R2 | BR-11002 | 150 | 100 | 1.360000 | 0.8972685 | 0.7387755 |
| R3 | BR-11002 | 150 | 100 | 1.440000 | 0.9555658 | 0.6775510 |
| R4 | BR-11002 | 150 | 100 | 1.380000 | 0.9792118 | 0.7061224 |
| R1 | BR-11002 | 300 | 98 | 1.755102 | 1.6618510 | 0.4005102 |
| R2 | BR-11002 | 300 | 98 | 1.530612 | 1.1669527 | 0.4761905 |
| R3 | BR-11002 | 300 | 100 | 1.760000 | 1.5892751 | 0.4465306 |
| R4 | BR-11002 | 300 | 100 | 1.920000 | 1.7961182 | 0.3240816 |
| R1 | BR-11002 | 450 | 82 | 3.780488 | 2.8971467 | 0.1500000 |
| R2 | BR-11002 | 450 | 84 | 3.166667 | 2.6581963 | 0.1718931 |
| R3 | BR-11002 | 450 | 86 | 3.813954 | 2.9186325 | 0.1317829 |
| R4 | BR-11002 | 450 | 84 | 3.809524 | 2.8385464 | 0.1428571 |

Code

```
dt <- gsm %>% 
  mutate(strains = factor(strains
                         , levels = c("Control", "BR-11001", "BR-11002")
                         , ordered = TRUE))

rs <- xfun::cache_rds({
  
  traits <- names(dt)[4:length(dt)]
  # trait <- "grp" 
  
  results <- traits %>% set_names() %>% map( \(trait) {
    
    # Liberar memoria
    gc()
    
    # Definir modelo
    mdf <- paste(trait, "0 + strains*nacl + (1|repetition)", sep = " ~ ") 
    
    # Mostrar qué rasgo se analiza
    cat("\n\n## Análisis para: ", trait, "\n\n")
    
    
    cat("\n\n### Diagnostico de modelo\n\n")
    
    # Remover outliers (asumo que tienes esta función personalizada)
    md <- dt %>% 
      remove_outliers(as.formula(mdf),
                      plot_diag = TRUE,
                      drop_na = TRUE)
    
    md$diagplot %>% print()
    
    if (nrow(md$outliers) > 0) {
    cat("> Outliers detectados\n")
    md$outliers %>% kable() %>% print()
    } else {
    cat("> No se detectaron outliers\n")
    }
    
    mdc <- md$model$raw
    
    cat("\n\n### ANOVA tipo III\n\n")
    
    Anova(mdc, type = 3, test.statistic = "F") %>%  print()
    
    cat("\n\n### Comparaciones múltiples (Emmeans)\n\n")
    
    # Comparaciones múltiples into the groups
    mc_in <- emmeans(mdc, ~ strains|nacl, type = "response") %>% 
      cld(Letters = letters, reversed = TRUE) %>% 
      mutate(across(".group", trimws)) %>% 
      mutate(across(".group", tolower)) %>% 
      rename(sig_in = ".group")
    
    mc_ou <- emmeans(mdc, ~ nacl|strains, type = "response") %>% 
      cld(Letters = letters, reversed = TRUE) %>% 
      mutate(across(".group", trimws)) %>% 
      mutate(across(".group", toupper)) %>% 
      rename(sig_ou = ".group")
    
    mc <- merge(mc_in, mc_ou) %>% 
      unite(col = "sig", c("sig_in", "sig_ou"), sep = "")
    
    mc %>% kable() %>% print()
    
    cat("\n\n### Gráfico resumen\n\n")
    
    plot <- mc %>% 
      plot_smr(type = "line",
               x = "nacl",
               y = "emmean"
               , group = "strains"
               , ylab = trait
               , sig = "sig"
               , error = "SE") +
      scale_color_manual(values = c("#fbb4ae", "#b3cde3", "#ccebc5"))
    
    plot %>% print()
    
    list(mc = mc,
         plot = plot
         )
  })
  
  results
  
}, file = "result.rds", rerun = T)
## 
## 
## ## Análisis para:  grp 
## 
## 
## 
## ### Diagnostico de modelo
```

```
## > Outliers detectados
## 
## 
## |   |index |strains  |nacl |repetition | grp| resi|         res_MAD| rawp.BHStud| adjp| bholm|out_flag |
## |:--|:-----|:--------|:----|:----------|---:|----:|---------------:|-----------:|----:|-----:|:--------|
## |9  |9     |Control  |300  |R1         |  96|  0.5|  11865766925951|           0|    0|     0|OUTLIER  |
## |10 |10    |Control  |300  |R2         |  94| -1.5| -35597300777855|           0|    0|     0|OUTLIER  |
## |11 |11    |Control  |300  |R3         |  96|  0.5|  11865766925951|           0|    0|     0|OUTLIER  |
## |12 |12    |Control  |300  |R4         |  96|  0.5|  11865766925951|           0|    0|     0|OUTLIER  |
## |13 |13    |Control  |450  |R1         |  56|  3.5|  83060368481662|           0|    0|     0|OUTLIER  |
## |14 |14    |Control  |450  |R2         |  52| -0.5| -11865766925952|           0|    0|     0|OUTLIER  |
## |15 |15    |Control  |450  |R3         |  52| -0.5| -11865766925952|           0|    0|     0|OUTLIER  |
## |16 |16    |Control  |450  |R4         |  50| -2.5| -59328834629758|           0|    0|     0|OUTLIER  |
## |25 |25    |BR-11001 |300  |R1         |  98| -0.5| -11865766925952|           0|    0|     0|OUTLIER  |
## |26 |26    |BR-11001 |300  |R2         | 100|  1.5|  35597300777855|           0|    0|     0|OUTLIER  |
## |27 |27    |BR-11001 |300  |R3         |  98| -0.5| -11865766925952|           0|    0|     0|OUTLIER  |
## |28 |28    |BR-11001 |300  |R4         |  98| -0.5| -11865766925952|           0|    0|     0|OUTLIER  |
## |29 |29    |BR-11001 |450  |R1         |  76| -2.5| -59328834629758|           0|    0|     0|OUTLIER  |
## |30 |30    |BR-11001 |450  |R2         |  78| -0.5| -11865766925951|           0|    0|     0|OUTLIER  |
## |31 |31    |BR-11001 |450  |R3         |  80|  1.5|  35597300777855|           0|    0|     0|OUTLIER  |
## |32 |32    |BR-11001 |450  |R4         |  80|  1.5|  35597300777855|           0|    0|     0|OUTLIER  |
## |41 |41    |BR-11002 |300  |R1         |  98| -1.0| -23731533851904|           0|    0|     0|OUTLIER  |
## |42 |42    |BR-11002 |300  |R2         |  98| -1.0| -23731533851904|           0|    0|     0|OUTLIER  |
## |43 |43    |BR-11002 |300  |R3         | 100|  1.0|  23731533851903|           0|    0|     0|OUTLIER  |
## |44 |44    |BR-11002 |300  |R4         | 100|  1.0|  23731533851903|           0|    0|     0|OUTLIER  |
## |45 |45    |BR-11002 |450  |R1         |  82| -2.0| -47463067703807|           0|    0|     0|OUTLIER  |
## |47 |47    |BR-11002 |450  |R3         |  86|  2.0|  47463067703807|           0|    0|     0|OUTLIER  |
## 
## 
## ### ANOVA tipo III
## 
## Analysis of Deviance Table (Type III Wald F tests with Kenward-Roger df)
## 
## Response: grp
##                     F Df Df.res                Pr(>F)    
## strains      29824.52  3 33.245 < 0.00000000000000022 ***
## nacl          1719.33  3 33.000 < 0.00000000000000022 ***
## strains:nacl   199.08  6 33.000 < 0.00000000000000022 ***
## ---
## Signif. codes:  0 '***' 0.001 '**' 0.01 '*' 0.05 '.' 0.1 ' ' 1
## 
## 
## ### Comparaciones múltiples (Emmeans)
## 
## 
## 
## |strains  |nacl | emmean|        SE| df| lower.CL|  upper.CL|sig |
## |:--------|:----|------:|---------:|--:|--------:|---------:|:---|
## |BR-11001 |0    |  100.0| 0.5773503| 36| 98.82908| 101.17092|aA  |
## |BR-11001 |150  |  100.0| 0.5773503| 36| 98.82908| 101.17092|aA  |
## |BR-11001 |300  |   98.5| 0.5773503| 36| 97.32908|  99.67092|aA  |
## |BR-11001 |450  |   78.5| 0.5773503| 36| 77.32908|  79.67092|bB  |
## |BR-11002 |0    |  100.0| 0.5773503| 36| 98.82908| 101.17092|aA  |
## |BR-11002 |150  |  100.0| 0.5773503| 36| 98.82908| 101.17092|aA  |
## |BR-11002 |300  |   99.0| 0.5773503| 36| 97.82908| 100.17092|aA  |
## |BR-11002 |450  |   84.0| 0.5773503| 36| 82.82908|  85.17092|aB  |
## |Control  |0    |  100.0| 0.5773503| 36| 98.82908| 101.17092|aA  |
## |Control  |150  |  100.0| 0.5773503| 36| 98.82908| 101.17092|aA  |
## |Control  |300  |   95.5| 0.5773503| 36| 94.32908|  96.67092|bB  |
## |Control  |450  |   52.5| 0.5773503| 36| 51.32908|  53.67092|cC  |
## 
## 
## ### Gráfico resumen
```

```
## 
## 
## ## Análisis para:  mgt 
## 
## 
## 
## ### Diagnostico de modelo
```

```
## > Outliers detectados
## 
## 
## |   |index |strains  |nacl |repetition |      mgt|       resi|   res_MAD| rawp.BHStud|           adjp|     bholm|out_flag |
## |:--|:-----|:--------|:----|:----------|--------:|----------:|---------:|-----------:|--------------:|---------:|:--------|
## |15 |15    |Control  |450  |R3         | 6.115385|  0.3376700|  3.692582|   0.0002220| 0.000221988925| 0.0104335|OUTLIER  |
## |46 |46    |BR-11002 |450  |R2         | 3.166667| -0.4404369| -4.816386|   0.0000015| 0.000001461813| 0.0000702|OUTLIER  |
## 
## 
## ### ANOVA tipo III
## 
## Analysis of Deviance Table (Type III Wald F tests with Kenward-Roger df)
## 
## Response: mgt
##                     F Df Df.res                Pr(>F)    
## strains       219.659  3 28.942 < 0.00000000000000022 ***
## nacl         1555.737  3 33.000 < 0.00000000000000022 ***
## strains:nacl   39.203  6 33.000    0.0000000000001223 ***
## ---
## Signif. codes:  0 '***' 0.001 '**' 0.01 '*' 0.05 '.' 0.1 ' ' 1
## 
## 
## ### Comparaciones múltiples (Emmeans)
## 
## 
## 
## |strains  |nacl |   emmean|       SE|       df| lower.CL| upper.CL|sig |
## |:--------|:----|--------:|--------:|--------:|--------:|--------:|:---|
## |BR-11001 |0    | 1.150000| 0.075536| 33.83642| 0.996465| 1.303535|aD  |
## |BR-11001 |150  | 1.700000| 0.075536| 33.83642| 1.546465| 1.853535|aC  |
## |BR-11001 |300  | 2.125816| 0.075536| 33.83642| 1.972281| 2.279351|bB  |
## |BR-11001 |450  | 5.216262| 0.075536| 33.83642| 5.062727| 5.369797|bA  |
## |BR-11002 |0    | 1.110000| 0.075536| 33.83642| 0.956465| 1.263535|aD  |
## |BR-11002 |150  | 1.405000| 0.075536| 33.83642| 1.251465| 1.558535|bC  |
## |BR-11002 |300  | 1.741429| 0.075536| 33.83642| 1.587894| 1.894964|cB  |
## |BR-11002 |450  | 3.642658| 0.075536| 33.83642| 3.489123| 3.796193|cA  |
## |Control  |0    | 1.355000| 0.075536| 33.83642| 1.201465| 1.508535|aD  |
## |Control  |150  | 1.750000| 0.075536| 33.83642| 1.596465| 1.903535|aC  |
## |Control  |300  | 2.459774| 0.075536| 33.83642| 2.306239| 2.613309|aB  |
## |Control  |450  | 5.742033| 0.075536| 33.83642| 5.588498| 5.895568|aA  |
## 
## 
## ### Gráfico resumen
```

```
## 
## 
## ## Análisis para:  unc 
## 
## 
## 
## ### Diagnostico de modelo
```

```
## > No se detectaron outliers
## 
## 
## ### ANOVA tipo III
## 
## Analysis of Deviance Table (Type III Wald F tests with Kenward-Roger df)
## 
## Response: unc
##                     F Df Df.res                Pr(>F)    
## strains       65.2786  3 23.393      0.00000000001624 ***
## nacl         806.2880  3 33.000 < 0.00000000000000022 ***
## strains:nacl   2.9991  6 33.000               0.01887 *  
## ---
## Signif. codes:  0 '***' 0.001 '**' 0.01 '*' 0.05 '.' 0.1 ' ' 1
## 
## 
## ### Comparaciones múltiples (Emmeans)
## 
## 
## 
## |strains  |nacl |    emmean|        SE|       df|  lower.CL|  upper.CL|sig |
## |:--------|:----|---------:|---------:|--------:|---------:|---------:|:---|
## |BR-11001 |0    | 0.5316838| 0.0649248| 26.43367| 0.3983355| 0.6650321|bD  |
## |BR-11001 |150  | 1.2286803| 0.0649248| 26.43367| 1.0953321| 1.3620286|aC  |
## |BR-11001 |300  | 1.6943196| 0.0649248| 26.43367| 1.5609713| 1.8276679|aB  |
## |BR-11001 |450  | 2.9065733| 0.0649248| 26.43367| 2.7732250| 3.0399216|aA  |
## |BR-11002 |0    | 0.4961936| 0.0649248| 26.43367| 0.3628453| 0.6295419|bD  |
## |BR-11002 |150  | 0.9185542| 0.0649248| 26.43367| 0.7852059| 1.0519025|bC  |
## |BR-11002 |300  | 1.5535492| 0.0649248| 26.43367| 1.4202010| 1.6868975|aB  |
## |BR-11002 |450  | 2.8281305| 0.0649248| 26.43367| 2.6947822| 2.9614788|aA  |
## |Control  |0    | 0.7738005| 0.0649248| 26.43367| 0.6404522| 0.9071488|aD  |
## |Control  |150  | 1.3523896| 0.0649248| 26.43367| 1.2190414| 1.4857379|aC  |
## |Control  |300  | 1.6980144| 0.0649248| 26.43367| 1.5646661| 1.8313627|aB  |
## |Control  |450  | 2.8561455| 0.0649248| 26.43367| 2.7227973| 2.9894938|aA  |
## 
## 
## ### Gráfico resumen
```

```
## 
## 
## ## Análisis para:  syn 
## 
## 
## 
## ### Diagnostico de modelo
```

```
## > No se detectaron outliers
## 
## 
## ### ANOVA tipo III
## 
## Analysis of Deviance Table (Type III Wald F tests with Kenward-Roger df)
## 
## Response: syn
##                      F Df Df.res                Pr(>F)    
## strains      1743.0005  3 23.735 < 0.00000000000000022 ***
## nacl         1196.5978  3 33.000 < 0.00000000000000022 ***
## strains:nacl    6.1427  6 33.000             0.0002109 ***
## ---
## Signif. codes:  0 '***' 0.001 '**' 0.01 '*' 0.05 '.' 0.1 ' ' 1
## 
## 
## ### Comparaciones múltiples (Emmeans)
## 
## 
## 
## |strains  |nacl |    emmean|        SE|       df|  lower.CL|  upper.CL|sig |
## |:--------|:----|---------:|---------:|--------:|---------:|---------:|:---|
## |BR-11001 |0    | 0.8293878| 0.0161694| 27.01915| 0.7962120| 0.8625635|aA  |
## |BR-11001 |150  | 0.6267347| 0.0161694| 27.01915| 0.5935589| 0.6599105|bB  |
## |BR-11001 |300  | 0.3208248| 0.0161694| 27.01915| 0.2876491| 0.3540006|bC  |
## |BR-11001 |450  | 0.1315092| 0.0161694| 27.01915| 0.0983334| 0.1646850|aD  |
## |BR-11002 |0    | 0.8012245| 0.0161694| 27.01915| 0.7680487| 0.8344003|aA  |
## |BR-11002 |150  | 0.7157143| 0.0161694| 27.01915| 0.6825385| 0.7488901|aB  |
## |BR-11002 |300  | 0.4118282| 0.0161694| 27.01915| 0.3786525| 0.4450040|aC  |
## |BR-11002 |450  | 0.1491333| 0.0161694| 27.01915| 0.1159575| 0.1823091|aD  |
## |Control  |0    | 0.7502041| 0.0161694| 27.01915| 0.7170283| 0.7833799|bA  |
## |Control  |150  | 0.5695918| 0.0161694| 27.01915| 0.5364161| 0.6027676|cB  |
## |Control  |300  | 0.3441644| 0.0161694| 27.01915| 0.3109886| 0.3773401|bC  |
## |Control  |450  | 0.1170726| 0.0161694| 27.01915| 0.0838969| 0.1502484|aD  |
## 
## 
## ### Gráfico resumen
```

Code

```
plots <- list(rs$grp$plot +
                scale_y_continuous(limits = c(50, 110)
                                  , breaks = seq(0, 200, by = 10)) +
                labs(x = ""
                     , y = "Germination percentage (%)"
                     ) +
                theme(legend.position = "none"
                      , axis.text.x = element_blank()
                      , strip.text = element_blank()
                      )
              , rs$mgt$plot +
                scale_y_continuous(limits = c(1, 6)
                                  , breaks = seq(0, 200, by = 1)) +
                labs(x = ""
                     , y = "Mean germination time (days)"
                     ) +
                theme(legend.position = "none"
                      , axis.text.x = element_blank()
                      , strip.text = element_blank()
                      )
              , rs$unc$plot +
                scale_y_continuous(limits = c(0.25, 3.25)
                                  , breaks = seq(0, 100, by = 0.5)) +
                labs(x = "NaCl (mM)"
                     , y = "Germination uncertainty"
                     ) +
                theme(legend.position = "none"
                      )
              , rs$syn$plot +
                scale_y_continuous(limits = c(0, 1)
                                  , breaks = seq(0, 100, by = 0.25)) +
                labs(x = "NaCl (mM)"
                     , y = "Germination synchrony"
                     ) +
                theme(legend.position = "none"
                      , axis.text.y = element_text(angle = 90)
                      )
              )

# extraer leyenda
legend <- get_plot_component(rs$grp$plot +
                               theme(legend.position = "top"
                                     , legend.direction = "horizontal")
                             , "guide-box", return_all = T)

fgrids <- plots %>% 
  cowplot::plot_grid(plotlist = ., ncol = 2, labels = "auto") 

export <-  list(legend[[4]], fgrids) %>% 
  cowplot::plot_grid(plotlist = .
                     , ncol = 1
                     , rel_heights = c(0.1, 1))
  
plot <- export %>% 
  ggsave(plot = ., "manuscript/Figure-03.jpg", width = 20, height = 18
         , units = "cm")

plot %>% include_graphics()
```

Code

```
export %>% 
  ggsave(plot = ., "manuscript/Figure-03.eps", width = 20, height = 18
         , units = "cm")
```

## 4.3 To quantify seedling growth by measuring shoot and root length and dry biomass in inoculated versus non-inoculated treatments under saline and non-saline conditions

Code

```
dtx <- growth %>% 
  mutate(strains = factor(strains
                         , levels = c("Control", "BR-11001", "BR-11002")
                         , ordered = TRUE)) %>% 
  merge(. , gsm) %>% 
  mutate(svi = (shoot_length + root_length)*grp)


# trait <- "shoot_length" 

rs <- xfun::cache_rds({
  
  traits <- names(dtx)[4:length(dtx)]
  
  results <- traits %>% set_names() %>% map( \(trait) {
    
    # Liberar memoria
    gc()
    
    # Definir modelo
    mdf <- paste(trait, "0 + strains*nacl + (1|repetition)", sep = " ~ ") 
    
    # Mostrar qué rasgo se analiza
    cat("\n\n## Análisis para: ", trait, "\n\n")
    
    
    cat("\n\n### Diagnostico de modelo\n\n")
    
    # Remover outliers (asumo que tienes esta función personalizada)
    md <- dtx %>% 
      remove_outliers(as.formula(mdf),
                      plot_diag = TRUE,
                      drop_na = TRUE)
    
    md$diagplot %>% print()
    
    if (nrow(md$outliers) > 0) {
    cat("> Outliers detectados\n")
    md$outliers %>% kable() %>% print()
    } else {
    cat("> No se detectaron outliers\n")
    }
    
    mdc <- md$model$clean
    
    cat("\n\n### ANOVA tipo III\n\n")
    
    Anova(mdc, type = 3, test.statistic = "F") %>%  print()
    
    cat("\n\n### Comparaciones múltiples (Emmeans)\n\n")
    
    # Comparaciones múltiples into the groups
    mc_in <- emmeans(mdc, ~ strains|nacl, type = "response") %>% 
      cld(Letters = letters, reversed = TRUE) %>% 
      mutate(across(".group", trimws)) %>% 
      mutate(across(".group", tolower)) %>% 
      rename(sig_in = ".group")
    
    mc_ou <- emmeans(mdc, ~ nacl|strains, type = "response") %>% 
      cld(Letters = letters, reversed = TRUE) %>% 
      mutate(across(".group", trimws)) %>% 
      mutate(across(".group", toupper)) %>% 
      rename(sig_ou = ".group")
    
    mc <- merge(mc_in, mc_ou) %>% 
      unite(col = "sig", c("sig_in", "sig_ou"), sep = "")
    
    mc %>% kable() %>% print()
    
    cat("\n\n### Gráfico resumen\n\n")
    
    plot <- mc %>% 
      plot_smr(type = "line",
               x = "nacl",
               y = "emmean"
               , group = "strains"
               , ylab = trait
               , sig = "sig"
               , error = "SE") +
      scale_color_manual(values = c("#fbb4ae", "#b3cde3", "#ccebc5"))
    
    plot %>% print()
    
    list(mc = mc,
         plot = plot
         )
  })
  
  results
  
}, file = "result.rds", rerun = T)
## 
## 
## ## Análisis para:  shoot_length 
## 
## 
## 
## ### Diagnostico de modelo
```

```
## > No se detectaron outliers
## 
## 
## ### ANOVA tipo III
## 
## Analysis of Deviance Table (Type III Wald F tests with Kenward-Roger df)
## 
## Response: shoot_length
##                     F Df Df.res                Pr(>F)    
## strains      51368.34  3 33.245 < 0.00000000000000022 ***
## nacl          6868.36  3 33.000 < 0.00000000000000022 ***
## strains:nacl    86.68  6 33.000 < 0.00000000000000022 ***
## ---
## Signif. codes:  0 '***' 0.001 '**' 0.01 '*' 0.05 '.' 0.1 ' ' 1
## 
## 
## ### Comparaciones múltiples (Emmeans)
## 
## 
## 
## |strains  |nacl |   emmean|        SE| df| lower.CL| upper.CL|sig |
## |:--------|:----|--------:|---------:|--:|--------:|--------:|:---|
## |BR-11001 |0    | 5.809725| 0.0244621| 36| 5.760114| 5.859337|bA  |
## |BR-11001 |150  | 5.797650| 0.0244621| 36| 5.748038| 5.847262|bA  |
## |BR-11001 |300  | 4.558875| 0.0244621| 36| 4.509264| 4.608486|bB  |
## |BR-11001 |450  | 3.062825| 0.0244621| 36| 3.013213| 3.112436|bC  |
## |BR-11002 |0    | 6.020650| 0.0244621| 36| 5.971038| 6.070261|aA  |
## |BR-11002 |150  | 6.033325| 0.0244621| 36| 5.983714| 6.082936|aA  |
## |BR-11002 |300  | 5.262625| 0.0244621| 36| 5.213013| 5.312237|aB  |
## |BR-11002 |450  | 3.959200| 0.0244621| 36| 3.909588| 4.008812|aC  |
## |Control  |0    | 4.770450| 0.0244621| 36| 4.720839| 4.820062|cA  |
## |Control  |150  | 4.727025| 0.0244621| 36| 4.677414| 4.776637|cA  |
## |Control  |300  | 4.136000| 0.0244621| 36| 4.086388| 4.185612|cB  |
## |Control  |450  | 2.110125| 0.0244621| 36| 2.060513| 2.159737|cC  |
## 
## 
## ### Gráfico resumen
```

```
## 
## 
## ## Análisis para:  root_length 
## 
## 
## 
## ### Diagnostico de modelo
```

```
## > No se detectaron outliers
## 
## 
## ### ANOVA tipo III
## 
## Analysis of Deviance Table (Type III Wald F tests with Kenward-Roger df)
## 
## Response: root_length
##                      F Df Df.res                Pr(>F)    
## strains      16443.532  3 31.751 < 0.00000000000000022 ***
## nacl          2541.001  3 33.000 < 0.00000000000000022 ***
## strains:nacl    86.177  6 33.000 < 0.00000000000000022 ***
## ---
## Signif. codes:  0 '***' 0.001 '**' 0.01 '*' 0.05 '.' 0.1 ' ' 1
## 
## 
## ### Comparaciones múltiples (Emmeans)
## 
## 
## 
## |strains  |nacl |   emmean|        SE|       df|  lower.CL|  upper.CL|sig |
## |:--------|:----|--------:|---------:|--------:|---------:|---------:|:---|
## |BR-11001 |0    | 3.316350| 0.0260871| 35.67545| 3.2634262| 3.3692738|bA  |
## |BR-11001 |150  | 3.299150| 0.0260871| 35.67545| 3.2462262| 3.3520738|bA  |
## |BR-11001 |300  | 2.493275| 0.0260871| 35.67545| 2.4403512| 2.5461988|bB  |
## |BR-11001 |450  | 2.097775| 0.0260871| 35.67545| 2.0448512| 2.1506988|bC  |
## |BR-11002 |0    | 3.934625| 0.0260871| 35.67545| 3.8817012| 3.9875488|aA  |
## |BR-11002 |150  | 3.983000| 0.0260871| 35.67545| 3.9300762| 4.0359238|aA  |
## |BR-11002 |300  | 3.457075| 0.0260871| 35.67545| 3.4041512| 3.5099988|aB  |
## |BR-11002 |450  | 2.543650| 0.0260871| 35.67545| 2.4907262| 2.5965738|aC  |
## |Control  |0    | 3.051550| 0.0260871| 35.67545| 2.9986262| 3.1044738|cA  |
## |Control  |150  | 2.999075| 0.0260871| 35.67545| 2.9461512| 3.0519988|cA  |
## |Control  |300  | 2.153075| 0.0260871| 35.67545| 2.1001512| 2.2059988|cB  |
## |Control  |450  | 0.933900| 0.0260871| 35.67545| 0.8809762| 0.9868238|cC  |
## 
## 
## ### Gráfico resumen
```

```
## 
## 
## ## Análisis para:  fresh_weight 
## 
## 
## 
## ### Diagnostico de modelo
```

```
## > No se detectaron outliers
## 
## 
## ### ANOVA tipo III
## 
## Analysis of Deviance Table (Type III Wald F tests with Kenward-Roger df)
## 
## Response: fresh_weight
##                    F Df Df.res                Pr(>F)    
## strains      2441756  3 33.245 < 0.00000000000000022 ***
## nacl          337881  3 33.000 < 0.00000000000000022 ***
## strains:nacl   23722  6 33.000 < 0.00000000000000022 ***
## ---
## Signif. codes:  0 '***' 0.001 '**' 0.01 '*' 0.05 '.' 0.1 ' ' 1
## 
## 
## ### Comparaciones múltiples (Emmeans)
## 
## 
## 
## |strains  |nacl |   emmean|        SE| df|  lower.CL|  upper.CL|sig |
## |:--------|:----|--------:|---------:|--:|---------:|---------:|:---|
## |BR-11001 |0    | 0.413850| 0.0002851| 36| 0.4132718| 0.4144282|bA  |
## |BR-11001 |150  | 0.414225| 0.0002851| 36| 0.4136468| 0.4148032|bA  |
## |BR-11001 |300  | 0.299875| 0.0002851| 36| 0.2992968| 0.3004532|bB  |
## |BR-11001 |450  | 0.257300| 0.0002851| 36| 0.2567218| 0.2578782|bC  |
## |BR-11002 |0    | 0.556675| 0.0002851| 36| 0.5560968| 0.5572532|aA  |
## |BR-11002 |150  | 0.556750| 0.0002851| 36| 0.5561718| 0.5573282|aA  |
## |BR-11002 |300  | 0.314775| 0.0002851| 36| 0.3141968| 0.3153532|aB  |
## |BR-11002 |450  | 0.300075| 0.0002851| 36| 0.2994968| 0.3006532|aC  |
## |Control  |0    | 0.343000| 0.0002851| 36| 0.3424218| 0.3435782|cA  |
## |Control  |150  | 0.342850| 0.0002851| 36| 0.3422718| 0.3434282|cA  |
## |Control  |300  | 0.257125| 0.0002851| 36| 0.2565468| 0.2577032|cB  |
## |Control  |450  | 0.214125| 0.0002851| 36| 0.2135468| 0.2147032|cC  |
## 
## 
## ### Gráfico resumen
```

```
## 
## 
## ## Análisis para:  dry_weight 
## 
## 
## 
## ### Diagnostico de modelo
```

```
## > No se detectaron outliers
## 
## 
## ### ANOVA tipo III
## 
## Analysis of Deviance Table (Type III Wald F tests with Kenward-Roger df)
## 
## Response: dry_weight
##                     F Df Df.res                Pr(>F)    
## strains      14802.65  3 33.245 < 0.00000000000000022 ***
## nacl          1129.13  3 33.000 < 0.00000000000000022 ***
## strains:nacl   173.93  6 33.000 < 0.00000000000000022 ***
## ---
## Signif. codes:  0 '***' 0.001 '**' 0.01 '*' 0.05 '.' 0.1 ' ' 1
## 
## 
## ### Comparaciones múltiples (Emmeans)
## 
## 
## 
## |strains  |nacl |   emmean|        SE| df|  lower.CL|  upper.CL|sig |
## |:--------|:----|--------:|---------:|--:|---------:|---------:|:---|
## |BR-11001 |0    | 0.032300| 0.0003048| 36| 0.0316818| 0.0329182|bA  |
## |BR-11001 |150  | 0.032475| 0.0003048| 36| 0.0318568| 0.0330932|bA  |
## |BR-11001 |300  | 0.027275| 0.0003048| 36| 0.0266568| 0.0278932|bB  |
## |BR-11001 |450  | 0.025775| 0.0003048| 36| 0.0251568| 0.0263932|bC  |
## |BR-11002 |0    | 0.049100| 0.0003048| 36| 0.0484818| 0.0497182|aA  |
## |BR-11002 |150  | 0.049150| 0.0003048| 36| 0.0485318| 0.0497682|aA  |
## |BR-11002 |300  | 0.030475| 0.0003048| 36| 0.0298568| 0.0310932|aB  |
## |BR-11002 |450  | 0.031550| 0.0003048| 36| 0.0309318| 0.0321682|aB  |
## |Control  |0    | 0.026375| 0.0003048| 36| 0.0257568| 0.0269932|cA  |
## |Control  |150  | 0.026350| 0.0003048| 36| 0.0257318| 0.0269682|cA  |
## |Control  |300  | 0.020850| 0.0003048| 36| 0.0202318| 0.0214682|cB  |
## |Control  |450  | 0.018500| 0.0003048| 36| 0.0178818| 0.0191182|cC  |
## 
## 
## ### Gráfico resumen
```

```
## 
## 
## ## Análisis para:  ms_porc 
## 
## 
## 
## ### Diagnostico de modelo
```

```
## > No se detectaron outliers
## 
## 
## ### ANOVA tipo III
## 
## Analysis of Deviance Table (Type III Wald F tests with Kenward-Roger df)
## 
## Response: ms_porc
##                     F Df Df.res                Pr(>F)    
## strains      7712.487  3 32.162 < 0.00000000000000022 ***
## nacl          226.923  3 33.000 < 0.00000000000000022 ***
## strains:nacl   12.429  6 33.000          0.0000002787 ***
## ---
## Signif. codes:  0 '***' 0.001 '**' 0.01 '*' 0.05 '.' 0.1 ' ' 1
## 
## 
## ### Comparaciones múltiples (Emmeans)
## 
## 
## 
## |strains  |nacl |    emmean|        SE|       df|  lower.CL|  upper.CL|sig |
## |:--------|:----|---------:|---------:|--------:|---------:|---------:|:---|
## |BR-11001 |0    |  7.804621| 0.0901902| 35.81973|  7.621675|  7.987568|bC  |
## |BR-11001 |150  |  7.839800| 0.0901902| 35.81973|  7.656854|  8.022746|bC  |
## |BR-11001 |300  |  9.094932| 0.0901902| 35.81973|  8.911986|  9.277878|bB  |
## |BR-11001 |450  | 10.017337| 0.0901902| 35.81973|  9.834391| 10.200283|bA  |
## |BR-11002 |0    |  8.820208| 0.0901902| 35.81973|  8.637262|  9.003155|aC  |
## |BR-11002 |150  |  8.827945| 0.0901902| 35.81973|  8.644999|  9.010891|aC  |
## |BR-11002 |300  |  9.681266| 0.0901902| 35.81973|  9.498320|  9.864212|aB  |
## |BR-11002 |450  | 10.513656| 0.0901902| 35.81973| 10.330710| 10.696602|aA  |
## |Control  |0    |  7.689417| 0.0901902| 35.81973|  7.506471|  7.872364|bC  |
## |Control  |150  |  7.685450| 0.0901902| 35.81973|  7.502504|  7.868397|bC  |
## |Control  |300  |  8.108541| 0.0901902| 35.81973|  7.925594|  8.291487|cB  |
## |Control  |450  |  8.639402| 0.0901902| 35.81973|  8.456456|  8.822348|cA  |
## 
## 
## ### Gráfico resumen
```

```
## 
## 
## ## Análisis para:  humidity_cont 
## 
## 
## 
## ### Diagnostico de modelo
```

```
## > No se detectaron outliers
## 
## 
## ### ANOVA tipo III
## 
## Analysis of Deviance Table (Type III Wald F tests with Kenward-Roger df)
## 
## Response: humidity_cont
##                     F Df Df.res                Pr(>F)    
## strains      11634347  3 33.245 < 0.00000000000000022 ***
## nacl          1686160  3 33.000 < 0.00000000000000022 ***
## strains:nacl   111712  6 33.000 < 0.00000000000000022 ***
## ---
## Signif. codes:  0 '***' 0.001 '**' 0.01 '*' 0.05 '.' 0.1 ' ' 1
## 
## 
## ### Comparaciones múltiples (Emmeans)
## 
## 
## 
## |strains  |nacl |   emmean|        SE| df|  lower.CL|  upper.CL|sig |
## |:--------|:----|--------:|---------:|--:|---------:|---------:|:---|
## |BR-11001 |0    | 0.381550| 0.0001198| 36| 0.3813071| 0.3817929|bA  |
## |BR-11001 |150  | 0.381750| 0.0001198| 36| 0.3815071| 0.3819929|bA  |
## |BR-11001 |300  | 0.272600| 0.0001198| 36| 0.2723571| 0.2728429|bB  |
## |BR-11001 |450  | 0.231525| 0.0001198| 36| 0.2312821| 0.2317679|bC  |
## |BR-11002 |0    | 0.507575| 0.0001198| 36| 0.5073321| 0.5078179|aA  |
## |BR-11002 |150  | 0.507600| 0.0001198| 36| 0.5073571| 0.5078429|aA  |
## |BR-11002 |300  | 0.284300| 0.0001198| 36| 0.2840571| 0.2845429|aB  |
## |BR-11002 |450  | 0.268525| 0.0001198| 36| 0.2682821| 0.2687679|aC  |
## |Control  |0    | 0.316625| 0.0001198| 36| 0.3163821| 0.3168679|cA  |
## |Control  |150  | 0.316500| 0.0001198| 36| 0.3162571| 0.3167429|cA  |
## |Control  |300  | 0.236275| 0.0001198| 36| 0.2360321| 0.2365179|cB  |
## |Control  |450  | 0.195625| 0.0001198| 36| 0.1953821| 0.1958679|cC  |
## 
## 
## ### Gráfico resumen
```

```
## 
## 
## ## Análisis para:  humidity_porc 
## 
## 
## 
## ### Diagnostico de modelo
```

```
## > No se detectaron outliers
## 
## 
## ### ANOVA tipo III
## 
## Analysis of Deviance Table (Type III Wald F tests with Kenward-Roger df)
## 
## Response: humidity_porc
##                       F Df Df.res                Pr(>F)    
## strains      987417.131  3 32.162 < 0.00000000000000022 ***
## nacl            226.923  3 33.000 < 0.00000000000000022 ***
## strains:nacl     12.429  6 33.000          0.0000002787 ***
## ---
## Signif. codes:  0 '***' 0.001 '**' 0.01 '*' 0.05 '.' 0.1 ' ' 1
## 
## 
## ### Comparaciones múltiples (Emmeans)
## 
## 
## 
## |strains  |nacl |   emmean|        SE|       df| lower.CL| upper.CL|sig |
## |:--------|:----|--------:|---------:|--------:|--------:|--------:|:---|
## |BR-11001 |0    | 92.19538| 0.0901902| 35.81973| 92.01243| 92.37832|aA  |
## |BR-11001 |150  | 92.16020| 0.0901902| 35.81973| 91.97725| 92.34315|aA  |
## |BR-11001 |300  | 90.90507| 0.0901902| 35.81973| 90.72212| 91.08801|bB  |
## |BR-11001 |450  | 89.98266| 0.0901902| 35.81973| 89.79972| 90.16561|bC  |
## |BR-11002 |0    | 91.17979| 0.0901902| 35.81973| 90.99685| 91.36274|bA  |
## |BR-11002 |150  | 91.17205| 0.0901902| 35.81973| 90.98911| 91.35500|bA  |
## |BR-11002 |300  | 90.31873| 0.0901902| 35.81973| 90.13579| 90.50168|cB  |
## |BR-11002 |450  | 89.48634| 0.0901902| 35.81973| 89.30340| 89.66929|cC  |
## |Control  |0    | 92.31058| 0.0901902| 35.81973| 92.12764| 92.49353|aA  |
## |Control  |150  | 92.31455| 0.0901902| 35.81973| 92.13160| 92.49750|aA  |
## |Control  |300  | 91.89146| 0.0901902| 35.81973| 91.70851| 92.07441|aB  |
## |Control  |450  | 91.36060| 0.0901902| 35.81973| 91.17765| 91.54354|aC  |
## 
## 
## ### Gráfico resumen
```

```
## 
## 
## ## Análisis para:  sod 
## 
## 
## 
## ### Diagnostico de modelo
```

```
## > No se detectaron outliers
## 
## 
## ### ANOVA tipo III
## 
## Analysis of Deviance Table (Type III Wald F tests with Kenward-Roger df)
## 
## Response: sod
##                     F Df Df.res                Pr(>F)    
## strains        869.82  3 11.003    0.0000000000002362 ***
## nacl         57039.15  3 33.000 < 0.00000000000000022 ***
## strains:nacl   310.48  6 33.000 < 0.00000000000000022 ***
## ---
## Signif. codes:  0 '***' 0.001 '**' 0.01 '*' 0.05 '.' 0.1 ' ' 1
## 
## 
## ### Comparaciones múltiples (Emmeans)
## 
## 
## 
## |strains  |nacl |   emmean|        SE|       df| lower.CL| upper.CL|sig |
## |:--------|:----|--------:|---------:|--------:|--------:|--------:|:---|
## |BR-11001 |0    | 26.78000| 0.5248219| 3.291193| 25.19035| 28.36965|aD  |
## |BR-11001 |150  | 39.41000| 0.5248219| 3.291193| 37.82035| 40.99965|bC  |
## |BR-11001 |300  | 53.43625| 0.5248219| 3.291193| 51.84660| 55.02590|bB  |
## |BR-11001 |450  | 63.35875| 0.5248219| 3.291193| 61.76910| 64.94840|bA  |
## |BR-11002 |0    | 26.67125| 0.5248219| 3.291193| 25.08160| 28.26090|aD  |
## |BR-11002 |150  | 42.10250| 0.5248219| 3.291193| 40.51285| 43.69215|aC  |
## |BR-11002 |300  | 57.90625| 0.5248219| 3.291193| 56.31660| 59.49590|aB  |
## |BR-11002 |450  | 68.11625| 0.5248219| 3.291193| 66.52660| 69.70590|aA  |
## |Control  |0    | 23.96375| 0.5248219| 3.291193| 22.37410| 25.55340|bD  |
## |Control  |150  | 36.23125| 0.5248219| 3.291193| 34.64160| 37.82090|cC  |
## |Control  |300  | 48.24000| 0.5248219| 3.291193| 46.65035| 49.82965|cB  |
## |Control  |450  | 56.25375| 0.5248219| 3.291193| 54.66410| 57.84340|cA  |
## 
## 
## ### Gráfico resumen
```

```
## 
## 
## ## Análisis para:  cat 
## 
## 
## 
## ### Diagnostico de modelo
```

```
## > No se detectaron outliers
## 
## 
## ### ANOVA tipo III
## 
## Analysis of Deviance Table (Type III Wald F tests with Kenward-Roger df)
## 
## Response: cat
##                     F Df Df.res                Pr(>F)    
## strains        392.73  3 11.136      0.00000000001432 ***
## nacl         26515.18  3 33.000 < 0.00000000000000022 ***
## strains:nacl   231.98  6 33.000 < 0.00000000000000022 ***
## ---
## Signif. codes:  0 '***' 0.001 '**' 0.01 '*' 0.05 '.' 0.1 ' ' 1
## 
## 
## ### Comparaciones múltiples (Emmeans)
## 
## 
## 
## |strains  |nacl |   emmean|        SE|       df| lower.CL| upper.CL|sig |
## |:--------|:----|--------:|---------:|--------:|--------:|--------:|:---|
## |BR-11001 |0    | 14.93625| 0.4490014| 3.456453| 13.60837| 16.26413|bD  |
## |BR-11001 |150  | 25.46625| 0.4490014| 3.456453| 24.13837| 26.79413|bC  |
## |BR-11001 |300  | 35.49500| 0.4490014| 3.456453| 34.16712| 36.82288|bB  |
## |BR-11001 |450  | 41.70000| 0.4490014| 3.456453| 40.37212| 43.02788|bA  |
## |BR-11002 |0    | 16.01250| 0.4490014| 3.456453| 14.68462| 17.34038|aD  |
## |BR-11002 |150  | 26.80125| 0.4490014| 3.456453| 25.47337| 28.12913|aC  |
## |BR-11002 |300  | 39.09125| 0.4490014| 3.456453| 37.76337| 40.41913|aB  |
## |BR-11002 |450  | 46.05875| 0.4490014| 3.456453| 44.73087| 47.38663|aA  |
## |Control  |0    | 14.15125| 0.4490014| 3.456453| 12.82337| 15.47913|cD  |
## |Control  |150  | 21.94750| 0.4490014| 3.456453| 20.61962| 23.27538|cC  |
## |Control  |300  | 31.12375| 0.4490014| 3.456453| 29.79587| 32.45163|cB  |
## |Control  |450  | 35.72500| 0.4490014| 3.456453| 34.39712| 37.05288|cA  |
## 
## 
## ### Gráfico resumen
```

```
## 
## 
## ## Análisis para:  apx 
## 
## 
## 
## ### Diagnostico de modelo
```

```
## > No se detectaron outliers
## 
## 
## ### ANOVA tipo III
## 
## Analysis of Deviance Table (Type III Wald F tests with Kenward-Roger df)
## 
## Response: apx
##                     F Df Df.res                Pr(>F)    
## strains       142.535  3 11.265        0.000000003101 ***
## nacl         8002.578  3 33.000 < 0.00000000000000022 ***
## strains:nacl   70.101  6 33.000 < 0.00000000000000022 ***
## ---
## Signif. codes:  0 '***' 0.001 '**' 0.01 '*' 0.05 '.' 0.1 ' ' 1
## 
## 
## ### Comparaciones múltiples (Emmeans)
## 
## 
## 
## |strains  |nacl |   emmean|        SE|       df|  lower.CL| upper.CL|sig |
## |:--------|:----|--------:|---------:|--------:|---------:|--------:|:---|
## |BR-11001 |0    | 10.84000| 0.5221022| 3.619693|  9.328325| 12.35167|aD  |
## |BR-11001 |150  | 17.74625| 0.5221022| 3.619693| 16.234575| 19.25792|bC  |
## |BR-11001 |300  | 25.33125| 0.5221022| 3.619693| 23.819575| 26.84292|bB  |
## |BR-11001 |450  | 29.64125| 0.5221022| 3.619693| 28.129575| 31.15292|bA  |
## |BR-11002 |0    | 10.75625| 0.5221022| 3.619693|  9.244575| 12.26792|aD  |
## |BR-11002 |150  | 19.40750| 0.5221022| 3.619693| 17.895825| 20.91917|aC  |
## |BR-11002 |300  | 28.38000| 0.5221022| 3.619693| 26.868325| 29.89167|aB  |
## |BR-11002 |450  | 33.04625| 0.5221022| 3.619693| 31.534575| 34.55792|aA  |
## |Control  |0    |  9.36000| 0.5221022| 3.619693|  7.848325| 10.87167|bD  |
## |Control  |150  | 15.19750| 0.5221022| 3.619693| 13.685825| 16.70917|cC  |
## |Control  |300  | 21.74500| 0.5221022| 3.619693| 20.233325| 23.25667|cB  |
## |Control  |450  | 25.58750| 0.5221022| 3.619693| 24.075825| 27.09917|cA  |
## 
## 
## ### Gráfico resumen
```

```
## 
## 
## ## Análisis para:  gpx 
## 
## 
## 
## ### Diagnostico de modelo
```

```
## > Outliers detectados
## 
## 
## |   |index |strains  |nacl |repetition |  gpx|       resi|   res_MAD| rawp.BHStud|         adjp|    bholm|out_flag |
## |:--|:-----|:--------|:----|:----------|----:|----------:|---------:|-----------:|------------:|--------:|:--------|
## |20 |20    |BR-11002 |0    |R4         | 7.74| -0.7986443| -3.515746|   0.0004385| 0.0004385205| 0.021049|OUTLIER  |
## 
## 
## ### ANOVA tipo III
## 
## Analysis of Deviance Table (Type III Wald F tests with Kenward-Roger df)
## 
## Response: gpx
##                     F Df Df.res                Pr(>F)    
## strains        65.977  3 10.985          0.0000002597 ***
## nacl         5923.719  3 32.001 < 0.00000000000000022 ***
## strains:nacl   69.612  6 32.001 < 0.00000000000000022 ***
## ---
## Signif. codes:  0 '***' 0.001 '**' 0.01 '*' 0.05 '.' 0.1 ' ' 1
## 
## 
## ### Comparaciones múltiples (Emmeans)
## 
## 
## 
## |strains  |nacl |    emmean|        SE|       df|  lower.CL|  upper.CL|sig |
## |:--------|:----|---------:|---------:|--------:|---------:|---------:|:---|
## |BR-11001 |0    |  7.933750| 0.5459773| 3.319524|  6.287096|  9.580404|aD  |
## |BR-11001 |150  | 11.718750| 0.5459773| 3.319524| 10.072096| 13.365404|bC  |
## |BR-11001 |300  | 17.838750| 0.5459773| 3.319524| 16.192096| 19.485404|bB  |
## |BR-11001 |450  | 20.861250| 0.5459773| 3.319524| 19.214596| 22.507904|bA  |
## |BR-11002 |0    |  7.615149| 0.5513238| 3.450493|  5.983326|  9.246973|abD |
## |BR-11002 |150  | 13.461250| 0.5459773| 3.319524| 11.814596| 15.107904|aC  |
## |BR-11002 |300  | 18.953750| 0.5459773| 3.319524| 17.307096| 20.600404|aB  |
## |BR-11002 |450  | 23.381250| 0.5459773| 3.319524| 21.734596| 25.027904|aA  |
## |Control  |0    |  7.148750| 0.5459773| 3.319524|  5.502096|  8.795404|bD  |
## |Control  |150  | 10.266250| 0.5459773| 3.319524|  8.619596| 11.912904|cC  |
## |Control  |300  | 14.838750| 0.5459773| 3.319524| 13.192096| 16.485404|cB  |
## |Control  |450  | 17.778750| 0.5459773| 3.319524| 16.132096| 19.425404|cA  |
## 
## 
## ### Gráfico resumen
```

```
## 
## 
## ## Análisis para:  grp 
## 
## 
## 
## ### Diagnostico de modelo
```

```
## > Outliers detectados
## 
## 
## |   |index |strains  |nacl |repetition | grp| resi|          res_MAD| rawp.BHStud| adjp| bholm|out_flag |
## |:--|:-----|:--------|:----|:----------|---:|----:|----------------:|-----------:|----:|-----:|:--------|
## |9  |9     |BR-11001 |300  |R1         |  98| -0.5|  -23731533851904|           0|    0|     0|OUTLIER  |
## |10 |10    |BR-11001 |300  |R2         | 100|  1.5|   71194601555710|           0|    0|     0|OUTLIER  |
## |11 |11    |BR-11001 |300  |R3         |  98| -0.5|  -23731533851904|           0|    0|     0|OUTLIER  |
## |12 |12    |BR-11001 |300  |R4         |  98| -0.5|  -23731533851904|           0|    0|     0|OUTLIER  |
## |13 |13    |BR-11001 |450  |R1         |  76| -2.5| -118657669259517|           0|    0|     0|OUTLIER  |
## |14 |14    |BR-11001 |450  |R2         |  78| -0.5|  -23731533851903|           0|    0|     0|OUTLIER  |
## |15 |15    |BR-11001 |450  |R3         |  80|  1.5|   71194601555710|           0|    0|     0|OUTLIER  |
## |16 |16    |BR-11001 |450  |R4         |  80|  1.5|   71194601555710|           0|    0|     0|OUTLIER  |
## |25 |25    |BR-11002 |300  |R1         |  98| -1.0|  -47463067703807|           0|    0|     0|OUTLIER  |
## |26 |26    |BR-11002 |300  |R2         |  98| -1.0|  -47463067703807|           0|    0|     0|OUTLIER  |
## |27 |27    |BR-11002 |300  |R3         | 100|  1.0|   47463067703807|           0|    0|     0|OUTLIER  |
## |28 |28    |BR-11002 |300  |R4         | 100|  1.0|   47463067703807|           0|    0|     0|OUTLIER  |
## |29 |29    |BR-11002 |450  |R1         |  82| -2.0|  -94926135407613|           0|    0|     0|OUTLIER  |
## |31 |31    |BR-11002 |450  |R3         |  86|  2.0|   94926135407614|           0|    0|     0|OUTLIER  |
## |41 |41    |Control  |300  |R1         |  96|  0.5|   23731533851903|           0|    0|     0|OUTLIER  |
## |42 |42    |Control  |300  |R2         |  94| -1.5|  -71194601555711|           0|    0|     0|OUTLIER  |
## |43 |43    |Control  |300  |R3         |  96|  0.5|   23731533851903|           0|    0|     0|OUTLIER  |
## |44 |44    |Control  |300  |R4         |  96|  0.5|   23731533851903|           0|    0|     0|OUTLIER  |
## |45 |45    |Control  |450  |R1         |  56|  3.5|  166120736963325|           0|    0|     0|OUTLIER  |
## |46 |46    |Control  |450  |R2         |  52| -0.5|  -23731533851903|           0|    0|     0|OUTLIER  |
## |47 |47    |Control  |450  |R3         |  52| -0.5|  -23731533851903|           0|    0|     0|OUTLIER  |
## |48 |48    |Control  |450  |R4         |  50| -2.5| -118657669259517|           0|    0|     0|OUTLIER  |
## 
## 
## ### ANOVA tipo III
## 
## Analysis of Deviance Table (Type III Wald F tests with Kenward-Roger df)
## 
## Response: grp
##                                                  F Df Df.res
## strains      89732522025707554560428464086064.0000  3 13.111
## nacl           674537293467640588642442006462.0000  2 16.361
## strains:nacl                                2.5243  2 16.003
##                           Pr(>F)    
## strains      <0.0000000000000002 ***
## nacl         <0.0000000000000002 ***
## strains:nacl              0.1115    
## ---
## Signif. codes:  0 '***' 0.001 '**' 0.01 '*' 0.05 '.' 0.1 ' ' 1
## 
## 
## ### Comparaciones múltiples (Emmeans)
## 
## 
## 
## |strains  |nacl | emmean| SE|       df| lower.CL| upper.CL|sig |
## |:--------|:----|------:|--:|--------:|--------:|--------:|:---|
## |BR-11001 |0    |    100|  0| 15.95945|      100|      100|aA  |
## |BR-11001 |150  |    100|  0| 15.95945|      100|      100|aA  |
## |BR-11001 |450  |       |   |         |         |         |    |
## |BR-11002 |0    |    100|  0| 15.95945|      100|      100|aA  |
## |BR-11002 |150  |    100|  0| 15.95945|      100|      100|aA  |
## |BR-11002 |450  |     84|  0| 18.78373|       84|       84|aB  |
## |Control  |0    |    100|  0| 15.95945|      100|      100|aA  |
## |Control  |150  |    100|  0| 15.95945|      100|      100|aA  |
## |Control  |450  |       |   |         |         |         |    |
## 
## 
## ### Gráfico resumen
```

```
## 
## 
## ## Análisis para:  mgt 
## 
## 
## 
## ### Diagnostico de modelo
```

```
## > Outliers detectados
## 
## 
## |   |index |strains  |nacl |repetition |      mgt|       resi|   res_MAD| rawp.BHStud|           adjp|     bholm|out_flag |
## |:--|:-----|:--------|:----|:----------|--------:|----------:|---------:|-----------:|--------------:|---------:|:--------|
## |30 |30    |BR-11002 |450  |R2         | 3.166667| -0.4404369| -4.816386|   0.0000015| 0.000001461813| 0.0000702|OUTLIER  |
## |47 |47    |Control  |450  |R3         | 6.115385|  0.3376700|  3.692582|   0.0002220| 0.000221988925| 0.0104335|OUTLIER  |
## 
## 
## ### ANOVA tipo III
## 
## Analysis of Deviance Table (Type III Wald F tests with Kenward-Roger df)
## 
## Response: mgt
##                     F Df Df.res                Pr(>F)    
## strains       592.599  3 31.233 < 0.00000000000000022 ***
## nacl         2874.590  3 31.176 < 0.00000000000000022 ***
## strains:nacl   50.111  6 31.279   0.00000000000001103 ***
## ---
## Signif. codes:  0 '***' 0.001 '**' 0.01 '*' 0.05 '.' 0.1 ' ' 1
## 
## 
## ### Comparaciones múltiples (Emmeans)
## 
## 
## 
## |strains  |nacl |   emmean|        SE| df| lower.CL| upper.CL|sig |
## |:--------|:----|--------:|---------:|--:|--------:|--------:|:---|
## |BR-11001 |0    | 1.150000| 0.0495327| 34| 1.049338| 1.250663|bD  |
## |BR-11001 |150  | 1.700000| 0.0495327| 34| 1.599338| 1.800663|aC  |
## |BR-11001 |300  | 2.125816| 0.0495327| 34| 2.025154| 2.226479|bB  |
## |BR-11001 |450  | 5.216262| 0.0495327| 34| 5.115599| 5.316924|bA  |
## |BR-11002 |0    | 1.110000| 0.0495327| 34| 1.009337| 1.210662|bD  |
## |BR-11002 |150  | 1.405000| 0.0495327| 34| 1.304337| 1.505662|bC  |
## |BR-11002 |300  | 1.741429| 0.0495327| 34| 1.640766| 1.842091|cB  |
## |BR-11002 |450  | 3.801322| 0.0580728| 34| 3.683304| 3.919340|cA  |
## |Control  |0    | 1.355000| 0.0495327| 34| 1.254338| 1.455663|aD  |
## |Control  |150  | 1.750000| 0.0495327| 34| 1.649337| 1.850663|aC  |
## |Control  |300  | 2.459774| 0.0495327| 34| 2.359111| 2.560436|aB  |
## |Control  |450  | 5.617582| 0.0580728| 34| 5.499564| 5.735601|aA  |
## 
## 
## ### Gráfico resumen
```

```
## 
## 
## ## Análisis para:  unc 
## 
## 
## 
## ### Diagnostico de modelo
```

```
## > No se detectaron outliers
## 
## 
## ### ANOVA tipo III
## 
## Analysis of Deviance Table (Type III Wald F tests with Kenward-Roger df)
## 
## Response: unc
##                     F Df Df.res                Pr(>F)    
## strains       65.2786  3 23.393      0.00000000001624 ***
## nacl         806.2880  3 33.000 < 0.00000000000000022 ***
## strains:nacl   2.9991  6 33.000               0.01887 *  
## ---
## Signif. codes:  0 '***' 0.001 '**' 0.01 '*' 0.05 '.' 0.1 ' ' 1
## 
## 
## ### Comparaciones múltiples (Emmeans)
## 
## 
## 
## |strains  |nacl |    emmean|        SE|       df|  lower.CL|  upper.CL|sig |
## |:--------|:----|---------:|---------:|--------:|---------:|---------:|:---|
## |BR-11001 |0    | 0.5316838| 0.0649248| 26.43367| 0.3983355| 0.6650321|bD  |
## |BR-11001 |150  | 1.2286803| 0.0649248| 26.43367| 1.0953321| 1.3620286|aC  |
## |BR-11001 |300  | 1.6943196| 0.0649248| 26.43367| 1.5609713| 1.8276679|aB  |
## |BR-11001 |450  | 2.9065733| 0.0649248| 26.43367| 2.7732250| 3.0399216|aA  |
## |BR-11002 |0    | 0.4961936| 0.0649248| 26.43367| 0.3628453| 0.6295419|bD  |
## |BR-11002 |150  | 0.9185542| 0.0649248| 26.43367| 0.7852059| 1.0519025|bC  |
## |BR-11002 |300  | 1.5535492| 0.0649248| 26.43367| 1.4202010| 1.6868975|aB  |
## |BR-11002 |450  | 2.8281305| 0.0649248| 26.43367| 2.6947822| 2.9614788|aA  |
## |Control  |0    | 0.7738005| 0.0649248| 26.43367| 0.6404522| 0.9071488|aD  |
## |Control  |150  | 1.3523896| 0.0649248| 26.43367| 1.2190414| 1.4857379|aC  |
## |Control  |300  | 1.6980144| 0.0649248| 26.43367| 1.5646661| 1.8313627|aB  |
## |Control  |450  | 2.8561455| 0.0649248| 26.43367| 2.7227973| 2.9894938|aA  |
## 
## 
## ### Gráfico resumen
```

```
## 
## 
## ## Análisis para:  syn 
## 
## 
## 
## ### Diagnostico de modelo
```

```
## > No se detectaron outliers
## 
## 
## ### ANOVA tipo III
## 
## Analysis of Deviance Table (Type III Wald F tests with Kenward-Roger df)
## 
## Response: syn
##                      F Df Df.res                Pr(>F)    
## strains      1743.0005  3 23.735 < 0.00000000000000022 ***
## nacl         1196.5978  3 33.000 < 0.00000000000000022 ***
## strains:nacl    6.1427  6 33.000             0.0002109 ***
## ---
## Signif. codes:  0 '***' 0.001 '**' 0.01 '*' 0.05 '.' 0.1 ' ' 1
## 
## 
## ### Comparaciones múltiples (Emmeans)
## 
## 
## 
## |strains  |nacl |    emmean|        SE|       df|  lower.CL|  upper.CL|sig |
## |:--------|:----|---------:|---------:|--------:|---------:|---------:|:---|
## |BR-11001 |0    | 0.8293878| 0.0161694| 27.01915| 0.7962120| 0.8625635|aA  |
## |BR-11001 |150  | 0.6267347| 0.0161694| 27.01915| 0.5935589| 0.6599105|bB  |
## |BR-11001 |300  | 0.3208248| 0.0161694| 27.01915| 0.2876491| 0.3540006|bC  |
## |BR-11001 |450  | 0.1315092| 0.0161694| 27.01915| 0.0983334| 0.1646850|aD  |
## |BR-11002 |0    | 0.8012245| 0.0161694| 27.01915| 0.7680487| 0.8344003|aA  |
## |BR-11002 |150  | 0.7157143| 0.0161694| 27.01915| 0.6825385| 0.7488901|aB  |
## |BR-11002 |300  | 0.4118282| 0.0161694| 27.01915| 0.3786525| 0.4450040|aC  |
## |BR-11002 |450  | 0.1491333| 0.0161694| 27.01915| 0.1159575| 0.1823091|aD  |
## |Control  |0    | 0.7502041| 0.0161694| 27.01915| 0.7170283| 0.7833799|bA  |
## |Control  |150  | 0.5695918| 0.0161694| 27.01915| 0.5364161| 0.6027676|cB  |
## |Control  |300  | 0.3441644| 0.0161694| 27.01915| 0.3109886| 0.3773401|bC  |
## |Control  |450  | 0.1170726| 0.0161694| 27.01915| 0.0838969| 0.1502484|aD  |
## 
## 
## ### Gráfico resumen
```

```
## 
## 
## ## Análisis para:  svi 
## 
## 
## 
## ### Diagnostico de modelo
```

```
## > No se detectaron outliers
## 
## 
## ### ANOVA tipo III
## 
## Analysis of Deviance Table (Type III Wald F tests with Kenward-Roger df)
## 
## Response: svi
##                     F Df Df.res                Pr(>F)    
## strains      31457.43  3 31.652 < 0.00000000000000022 ***
## nacl          7867.98  3 33.000 < 0.00000000000000022 ***
## strains:nacl    85.97  6 33.000 < 0.00000000000000022 ***
## ---
## Signif. codes:  0 '***' 0.001 '**' 0.01 '*' 0.05 '.' 0.1 ' ' 1
## 
## 
## ### Comparaciones múltiples (Emmeans)
## 
## 
## 
## |strains  |nacl |    emmean|       SE|       df| lower.CL|  upper.CL|sig |
## |:--------|:----|---------:|--------:|--------:|--------:|---------:|:---|
## |BR-11001 |0    |  912.6075| 4.911396| 35.63542| 902.6432|  922.5718|bA  |
## |BR-11001 |150  |  909.6800| 4.911396| 35.63542| 899.7157|  919.6443|bA  |
## |BR-11001 |300  |  694.6879| 4.911396| 35.63542| 684.7235|  704.6522|bB  |
## |BR-11001 |450  |  405.1010| 4.911396| 35.63542| 395.1367|  415.0654|bC  |
## |BR-11002 |0    |  995.5275| 4.911396| 35.63542| 985.5632| 1005.4918|aA  |
## |BR-11002 |150  | 1001.6325| 4.911396| 35.63542| 991.6682| 1011.5968|aA  |
## |BR-11002 |300  |  863.2957| 4.911396| 35.63542| 853.3314|  873.2600|aB  |
## |BR-11002 |450  |  546.1985| 4.911396| 35.63542| 536.2342|  556.1629|aC  |
## |Control  |0    |  782.2000| 4.911396| 35.63542| 772.2357|  792.1643|cA  |
## |Control  |150  |  772.6100| 4.911396| 35.63542| 762.6457|  782.5743|cA  |
## |Control  |300  |  600.6111| 4.911396| 35.63542| 590.6468|  610.5754|cB  |
## |Control  |450  |  159.7628| 4.911396| 35.63542| 149.7984|  169.7271|cC  |
## 
## 
## ### Gráfico resumen
```

Code

```
plots <- list(rs$shoot_length$plot +
                scale_y_continuous(limits = c(1, 7)
                                  , breaks = seq(0, 200, by = 1)) +
                labs(x = ""
                     , y = "Shoot length (cm)"
                     ) +
                theme(legend.position = "none"
                      , axis.text.x = element_blank()
                      , strip.text = element_blank()
                      )
              , rs$root_length$plot +
                scale_y_continuous(limits = c(0.5, 4.5)
                                  , breaks = seq(0, 200, by = 0.5)) +
                labs(x = ""
                     , y = "Root length (cm)"
                     ) +
                theme(legend.position = "none"
                      , axis.text.x = element_blank()
                      , strip.text = element_blank()
                      )
              , rs$dry_weight$plot +
                scale_y_continuous(limits = c(0.01, 0.06)
                                  , breaks = seq(0, 100, by = 0.01)) +
                labs(x = "NaCl (mM)"
                     , y = "Dry weight (g)"
                     ) +
                theme(legend.position = "none"
                      , axis.text.y = element_text(angle = 90)
                      )
              , rs$svi$plot +
                scale_y_continuous(limits = c(100, 1100)
                                  , breaks = seq(0, 2000, by = 200)) +
                labs(x = "NaCl (mM)"
                     , y = "Seedling vigor index"
                     ) +
                theme(legend.position = "none"
                      , axis.text.y = element_text(angle = 90)
                      )
              )

# extraer leyenda
legend <- get_plot_component(rs$shoot_length$plot +
                               theme(legend.position = "top"
                                     , legend.direction = "horizontal")
                             , "guide-box", return_all = T)

fgrids <- plots %>% 
  cowplot::plot_grid(plotlist = ., ncol = 2, labels = "auto") 

export <-  list(legend[[4]], fgrids) %>% 
  cowplot::plot_grid(plotlist = .
                     , ncol = 1
                     , rel_heights = c(0.1, 1))
  
plot <- export %>% 
  ggsave(plot = ., "manuscript/Figure-04.jpg", width = 22, height = 20
         , units = "cm")

plot %>% include_graphics()
```

Code

```
export %>% 
  ggsave(plot = ., "manuscript/Figure-04.eps", width = 22, height = 20
         , units = "cm")
```

## 4.4 To evaluate physiological responses to salinity through the activity of antioxidant enzymes (SOD, CAT, APX, GPX)

Code

```
plots <- list(rs$sod$plot +
                scale_y_continuous(limits = c(20, 70)
                                  , breaks = seq(0, 200, by = 10)) +
                labs(x = ""
                     , y = "Superoxide dismutase (SOD)"
                     ) +
                theme(legend.position = "none"
                      , axis.text.x = element_blank()
                      , strip.text = element_blank()
                      )
              , rs$cat$plot +
                scale_y_continuous(limits = c(10, 50)
                                  , breaks = seq(0, 200, by = 10)) +
                labs(x = ""
                     , y = "Catalase (CAT)"
                     ) +
                theme(legend.position = "none"
                      , axis.text.x = element_blank()
                      , strip.text = element_blank()
                      )
              , rs$apx$plot +
                scale_y_continuous(limits = c(7.5, 35)
                                  , breaks = seq(0, 100, by = 5)) +
                labs(x = "NaCl (mM)"
                     , y = "Ascorbate peroxidase (APX)"
                     ) +
                theme(legend.position = "none"
                      )
              , rs$gpx$plot +
                scale_y_continuous(limits = c(5, 25)
                                  , breaks = seq(0, 100, by = 5)) +
                labs(x = "NaCl (mM)"
                     , y = "Guaiacol peroxidase (GPX)"
                     ) +
                theme(legend.position = "none"
                      )
              )

# extraer leyenda
legend <- get_plot_component(rs$shoot_length$plot +
                               theme(legend.position = "top"
                                     , legend.direction = "horizontal")
                             , "guide-box", return_all = T)

fgrids <- plots %>% 
  cowplot::plot_grid(plotlist = ., ncol = 2, labels = "auto") 

export <-  list(legend[[4]], fgrids) %>% 
  cowplot::plot_grid(plotlist = .
                     , ncol = 1
                     , rel_heights = c(0.1, 1))
  
plot <- export %>% 
  ggsave(plot = ., "manuscript/Figure-05.jpg", width = 22, height = 20
         , units = "cm")

plot %>% include_graphics()
```

Code

```
export %>% 
  ggsave(plot = ., "manuscript/Figure-05.eps", width = 22, height = 20
         , units = "cm")
```

# 5 Interaction phenotipic and biochemical

Code

```
fbclean <- 1:length(rs) %>%  map( \(x) { 
  
  rs[[x]]$mc %>% 
    dplyr::select(1:emmean) %>% 
    dplyr::rename(!!names(rs)[x] := emmean)
  
  }) %>% 
  Reduce(function(...) merge(..., all = TRUE), .)
```

Code

```
dta <- fbclean %>% 
  dplyr::select(!c(fresh_weight
                   , ms_porc, humidity_cont, humidity_porc, 
                   , grp:syn
                   )) %>% 
  unite("treat", c(strains, nacl), remove = F, sep = "_") %>% 
  column_to_rownames("treat")

mv <- PCA(X = dta, scale.unit = T, quali.sup = 1:2, graph = F)

var <- plot.PCA(mv, choix = c("var")
                , autoLab = "yes"
                , cex = 1.2
                ) 

varcor <- mv$ind$coord %>% 
  data.frame() %>% 
  rownames_to_column("treat") %>% 
  separate(treat, c("G1", "G2"), remove = F, sep = "_")

test <- plot.PCA(mv
                , choix = c("ind")
                , autoLab = "yes"
                , cex = 0.8
                , invisible = "quali"
                , habillage = 1
                )

ind <- ggplot(varcor,
                aes(x = Dim.1,
                    y = Dim.2,
                    color = G1,
                    shape = G1,
                    label = treat)) +
  geom_point(size = 1.8) +
  ggrepel::geom_text_repel(size = 4, max.overlaps = Inf
                           ) +  
  scale_shape_manual(values = c(23, 22, 17, 24, 18, 21)) +  
  geom_hline(yintercept = 0, linetype = "dashed", color = "black") +
  geom_vline(xintercept = 0, linetype = "dashed", color = "black") +
  theme_classic(base_size = 9) +
  theme(
    legend.position = "bottom",
    legend.box = "horizontal",
    panel.border = element_rect(colour = "black", fill = NA, linewidth = 0.6),
    plot.title = element_text(hjust = 0.5, face = "bold") 
  ) +
  labs(
    title = "PCA graph of individuals",
    x = paste0("Dim 1 (", round(mv$eig[1, 2], 2), "%)"),
    y = paste0("Dim 2 (", round(mv$eig[2, 2], 2), "%)"),
    color = "Treatment",
    shape = "Treatment"
  ) +
  guides(
    color = guide_legend(nrow = 1, byrow = TRUE),
    shape = guide_legend(nrow = 1, byrow = TRUE)
  )

grid <- list(var, ind) %>% 
  plot_grid(plotlist = ., ncol = 2, labels = "AUTO", rel_widths = c(1, 1.3))

summary(mv, nbelements = Inf, nb.dec = 1)
## 
## Call:
## PCA(X = dta, scale.unit = T, quali.sup = 1:2, graph = F) 
## 
## 
## Eigenvalues
##                      Dim.1 Dim.2 Dim.3 Dim.4 Dim.5 Dim.6 Dim.7 Dim.8
## Variance               5.9   1.7   0.3   0.0   0.0   0.0   0.0   0.0
## % of var.             74.3  21.6   3.5   0.4   0.1   0.0   0.0   0.0
## Cumulative % of var.  74.3  96.0  99.5  99.9 100.0 100.0 100.0 100.0
## 
## Individuals
##                Dist   Dim.1  ctr cos2   Dim.2  ctr cos2   Dim.3  ctr cos2  
## BR-11001_0   |  2.9 |  -2.8 10.7  0.9 |  -0.9  3.5  0.1 |   0.2  1.7  0.0 |
## BR-11001_150 |  1.6 |  -1.5  3.0  0.9 |   0.3  0.4  0.0 |   0.5  6.5  0.1 |
## BR-11001_300 |  1.4 |   1.2  2.2  0.8 |   0.4  0.8  0.1 |   0.3  3.6  0.1 |
## BR-11001_450 |  3.3 |   3.2 14.8  1.0 |   0.2  0.1  0.0 |  -0.3  3.5  0.0 |
## BR-11002_0   |  3.8 |  -3.7 19.1  0.9 |   0.5  1.0  0.0 |  -1.0 26.7  0.1 |
## BR-11002_150 |  2.9 |  -2.1  6.3  0.5 |   1.9 18.0  0.4 |  -0.6 11.8  0.0 |
## BR-11002_300 |  2.2 |   0.8  0.8  0.1 |   1.8 16.3  0.7 |   0.8 19.4  0.1 |
## BR-11002_450 |  3.4 |   3.0 12.8  0.8 |   1.6 13.1  0.2 |  -0.2  0.8  0.0 |
## Control_0    |  2.9 |  -2.2  6.7  0.6 |  -1.9 17.9  0.4 |   0.2  0.7  0.0 |
## Control_150  |  1.5 |  -1.0  1.5  0.5 |  -1.0  4.9  0.4 |   0.3  3.3  0.0 |
## Control_300  |  1.6 |   1.2  2.0  0.6 |  -0.9  4.1  0.3 |   0.5  6.2  0.1 |
## Control_450  |  4.4 |   3.8 20.3  0.8 |  -2.0 19.9  0.2 |  -0.7 15.8  0.0 |
## 
## Variables
##                Dim.1  ctr cos2   Dim.2  ctr cos2   Dim.3  ctr cos2  
## shoot_length |  -0.9 13.1  0.8 |   0.4  9.8  0.2 |   0.2 12.9  0.0 |
## root_length  |  -0.8 11.9  0.7 |   0.5 15.4  0.3 |   0.1  2.2  0.0 |
## dry_weight   |  -0.7  7.3  0.4 |   0.6 21.7  0.4 |  -0.4 67.5  0.2 |
## sod          |   0.9 14.0  0.8 |   0.4  9.5  0.2 |   0.0  0.3  0.0 |
## cat          |   0.9 13.5  0.8 |   0.4 11.2  0.2 |   0.0  0.9  0.0 |
## apx          |   0.9 13.3  0.8 |   0.5 11.9  0.2 |   0.1  0.9  0.0 |
## gpx          |   0.9 13.5  0.8 |   0.4 11.1  0.2 |   0.0  0.1  0.0 |
## svi          |  -0.9 13.3  0.8 |   0.4  9.4  0.2 |   0.2 15.2  0.0 |
## 
## Supplementary categories
##                Dist   Dim.1 cos2 v.test   Dim.2 cos2 v.test   Dim.3 cos2 v.test
## Control      |  1.5 |   0.4  0.1    0.4 |  -1.5  0.9   -2.6 |   0.1  0.0    0.2
## BR-11001     |  0.2 |   0.1  0.1    0.1 |   0.0  0.0    0.0 |   0.2  0.7    0.8
## BR-11002     |  1.6 |  -0.5  0.1   -0.5 |   1.5  0.9    2.6 |  -0.2  0.0   -1.0
## 0            |  3.0 |  -2.9  0.9   -2.3 |  -0.8  0.1   -1.1 |  -0.2  0.0   -0.7
## 150          |  1.6 |  -1.5  0.9   -1.2 |   0.4  0.1    0.6 |   0.1  0.0    0.2
## 300          |  1.3 |   1.1  0.7    0.8 |   0.4  0.1    0.6 |   0.5  0.2    1.9
## 450          |  3.4 |   3.4  1.0    2.6 |  -0.1  0.0   -0.1 |  -0.4  0.0   -1.5
##               
## Control      |
## BR-11001     |
## BR-11002     |
## 0            |
## 150          |
## 300          |
## 450          |
pcainfo <- factoextra::get_pca_var(mv)
pcainfo$cor
##                   Dim.1     Dim.2       Dim.3         Dim.4        Dim.5
## shoot_length -0.8834916 0.4128814  0.19120332  0.1084074234 -0.009196021
## root_length  -0.8409049 0.5163196  0.07891082 -0.1408456601 -0.002664913
## dry_weight   -0.6571464 0.6135355 -0.43684452  0.0296108767  0.002858408
## sod           0.9131964 0.4051834  0.02772554  0.0057793927  0.025941027
## cat           0.8959367 0.4402613  0.04999076  0.0003493301  0.021261073
## apx           0.8892093 0.4541223  0.05164879  0.0073325239  0.007160950
## gpx           0.8972543 0.4376526  0.01862562 -0.0035068445 -0.054244469
## svi          -0.8905875 0.4029004  0.20720447  0.0136610840  0.010017527
pcainfo$contrib
##                  Dim.1     Dim.2      Dim.3         Dim.4      Dim.5
## shoot_length 13.129524  9.844814 12.9258200 35.8815440952  1.9580628
## root_length  11.894273 15.395514  2.2016099 60.5675516813  0.1644345
## dry_weight    7.263877 21.738837 67.4716012  2.6770446200  0.1891800
## sod          14.027249  9.481131  0.2717861  0.1019805819 15.5812042
## cat          13.502022 11.193809  0.8835817  0.0003725847 10.4663935
## apx          13.300016 11.909748  0.9431648  0.1641572445  1.1873207
## gpx          13.541763 11.061552  0.1226560  0.0375479392 68.1298774
## svi          13.341276  9.374594 15.1797803  0.5698012532  2.3235270

plot <- grid %>% 
  ggsave2(plot = ., "manuscript/Figure-06.eps"
          , width = 35
          , height = 15
          , units = "cm")

plot <- grid %>% 
  ggsave2(plot = ., "manuscript/Figure-06.jpg"
          , width = 35
          , height = 15
          , units = "cm")

include_graphics(plot)
```
